# Evolution of the Selenoproteome in *Helicobacter pylori* and Epsilonproteobacteria

**DOI:** 10.1093/gbe/evv177

**Published:** 2015-09-04

**Authors:** Pietro Cravedi, Giulia Mori, Frédéric Fischer, Riccardo Percudani

**Affiliations:** ^1^Department of Life Sciences, University of Parma, Italy; ^2^Unité Pathogenèse de Helicobacter, Département de Microbiologie, Institut Pasteur, ERL CNRS 3526, Paris, France

**Keywords:** genetic code, human pathogens, micronutrients, redox proteins, Selenocysteine

## Abstract

By competing for the acquisition of essential nutrients, *Helicobacter pylori* has the unique ability to persist in the human stomach, also causing nutritional insufficiencies in the host. Although the *H. pylori* genome apparently encodes selenocysteine synthase (SelA, HP1513), a key pyridoxal phosphate (PLP)-dependent enzyme for the incorporation of selenium into bacterial proteins, nothing is known about the use of this essential element in protein synthesis by this pathogen. We analyzed the evolution of the complete machinery for incorporation of selenium into proteins and the selenoproteome of several *H. pylori* strains and related Epsilonproteobacteria. Our searches identified the presence of selenoproteins—including the previously unknown DUF466 family—in various Epsilonproteobacteria, but not in *H. pylori*. We found that a complete system for selenocysteine incorporation was present in the Helicobacteriaceae ancestor and has been recently lost before the split of *Helicobacter acinonychis* and *H. pylori*. Our results indicate that *H. pylori*, at variance with other gastric and enterohepatic *Helicobacter*, does not use selenocysteine in protein synthesis and does not use selenium for tRNA wobble base modification. However, *selA* has survived as a functional gene, having lost the domain for the binding of selenocysteine tRNA, but maintaining the ability to bind the PLP cofactor. The evolutionary modifications described for the SelA protein of *H. pylori* find parallels in other bacterial and archaeal species, suggesting that an alternative enzymatic function is hidden in many proteins annotated as selenocysteinyl-tRNA synthase.

## Introduction

As the sole organism able to persistently colonize the human stomach (Marshall and Warren 1984), *Helicobacter pylori* has direct access to the nutrients introduced with the diet. The utilization of micronutrients is a major aspect of the bacterium pathogenesis for the key role of metalloenzymes (particularly [Ni]-urease and [NiFe]-hydrogenase) in the adaptation to the gastric niche (Maier et al. 1996; Ha et al. 2001; De Reuse et al. 2013), and the alteration of the host homeostasis caused by the infection (Barabino 2002; Lahner et al. 2012). The most studied example is the association of *H. pylori* infection with sideropenic anemia in children. The physiopathologic mechanism for this iron deficiency is thought to be multifactorial, including impaired absorption due to decreased acid secretion and active sequestration of the essential metal ion by the pathogen (Barabino 2002).

Selenium is a nonmetal micronutrient essential for human health (Rayman 2012). The necessity of selenium derives from its incorporation as selenocysteine (Sec) into a number of redox proteins (Kryukov et al. 2003) involved in physiological processes, such as aging, immune function, and reproduction (Ursini et al. 1999; Martin-Romero et al. 2001). There is scarce information on the role of selenium in *H. pylori* and its interaction with the host. No differences have been observed in circulating selenium between infected and noninfected individuals (Toyonaga et al. 2000; Ustündağ et al. 2001), but there is circumstantial evidence that selenium significantly increases in gastric tissue during *H. pylori* infection to return to normal levels after eradication (Ustündağ et al. 2001). Intriguingly, the *H. pylori* genome (Tomb et al. 1997) encodes a protein annotated as l-seryl-tRNA^Sec^ selenium transferase (selenocysteine synthase, SelA), suggesting a possible role for selenium and Sec in this bacterium.

SelA is a pyridoxal phosphate (PLP)-dependent enzyme (Itoh et al. 2013) catalyzing in bacteria the formation of selenocysteinyl-tRNA starting from an UGA decoding tRNA^Sec ^(SelC) charged with serine and selenophosphate, the product of the enzyme selenophosphate synthetase (SelD). Together with SelB, a selenocysteinyl-tRNA-specific translation factor, SelA, SelC, and SelD are components of the bacterial Sec-decoding trait, allowing the incorporation of Sec at specific UGA (opal) codons followed by Sec insertion sequence (SECIS) elements (Zinoni et al. 1987, 1990; Kryukov and Gladyshev 2004). As SelA homologs can be found in organisms lacking the Sec-decoding trait (Romero et al. 2005; Zhang et al. 2006), the presence of this gene does not imply that *H. pylori* uses Sec for protein synthesis. Although *H. pylori* SelA (HpSelA) is apparently orthologous to experimentally validated SelA, other Sel components and selenoproteins were not identified in the genome, raising questions about the origin and role of the *selA* gene in *H. pylori* (Romero et al. 2005).

The recent sequencing of numerous *H. pylori *strains (Ahmed et al. 2013), different gastric and enterohepatic *Helicobacter* species (Flahou et al. 2013) and related ε-proteobacteria genera (Lefébure et al. 2010), provides the opportunity for detailed examination of the evolution of the Sec-decoding trait and the selenoproteome in this group of bacteria. As selenoproteins and genes of the Sel machinery, such as *selC*, can be easily missing in genome annotations (Lowe and Eddy 1997; Castellano et al. 2008), we made use of a dedicated bioinformatics analysis for the identification of *sel* genes and genes encoding selenoproteins. This analysis provided evidence for the existence in gastric *Helicobacter* of an Sec-decoding trait that was lost in the common ancestor of *H. pylori* and *H. acinonychis*. Consistently, known and novel selenoproteins (including an unprecedented example of carboxyl-terminal Sec) were identified in Helicobacteriaceae, but not in *H. pylori* and *H. acinonychis. *Changes in the selection pressure acting on the *selA* gene during the evolution of gastric *Helicobacter* were found to correlate with modifications in the structural organization of the SelA protein. The loss of a small tRNA^Sec^ binding domain at the protein N terminus was identified as the hallmark of SelA homologs with a function different from selenocysteinyl-tRNA^Sec^ biosynthesis.

## Materials and Methods

### Species and Genes Phylogeny

The species tree was based on a rooted maximum-likelihood phylogeny of 454 phylum level phylogenetic markers for ε-proteobacteria (Wang and Wu 2013). The tree was processed in the R software environment using functions of the Ape library (Popescu et al. 2012). Tips were purged to include the genomes selected for the analysis with the “drop.tip” function, and the resulting tree was rendered ultrametric with the “chronos” function. Traits of gene presence/absence were plotted alongside the tips (see fig. 3) using the “table.phylo4d” function of the Adephylo package (Jombart and Dray 2010). The phylogenetic tree of SelA proteins was obtained using the maximum-likelihood method implemented in the RaxML program ver. 7.7.8 (Stamatakis 2006) using the PROTCATGTR amino acid substitution model. The SelA tree was compared with the species tree using the Ape “cophyplot” function. The prokaryotic SelA tree (see fig. 5) was rendered using Figtree (http://tree.bio.ed.ac.uk/software/figtree/).

### Identification of Sel Proteins and Genes Encoding Selenoproteins

Complete prokaryotic genomes were downloaded from the NCBI (National Center for Biotechnology Information) ftp site; ε-proteobacteria genomes considered for the analysis were selected from genome report files based on the availability of whole-genome phylogeny (Wang and Wu 2013) and clustered using a Genomic Similarity Score (GSSa) = 0.95 (Moreno-Hagelsieb et al. 2013). Proteins of the Sel machinery were identified by homology using annotated sequences from *Campylobacter jejuni*. Bona fide MnmH and YedF proteins from *Sulfurimonas autotrophica* and *Campylobacter lari*, encoded in Sel operons, were used as reference sequences to search proteins involved in selenium metabolism. Searches were conducted on both complete proteomes with BLASTp (*E* < 10^−^^10^) and translated genomes with tBLASTn (*E* < 10^−^^10^). To discriminate between equivalent and nonequivalent homologs, known paralogous proteins (e.g., EF-Tu) were included in the search. The BLAST output was parsed with a custom Perl script to obtain the results reported in supplementary table S1, Supplementary Material online.

Proteins containing an Sec UGA codon were identified by homology with a set of 1,022 annotated selenoproteins downloaded from the database of Trace Element Utilization (dbTEU; http://gladyshevlab.org/trace_element/). Annotated selenoproteins were used to query the genomes set with tBLASTn (*E* < 10^−^^6^). The top-scoring alignment of each DNA locus was parsed to identify the codon (either TGA or TGY) corresponding to an Sec in the query sequence, as reported in supplementary table S3, Supplementary Material online. Coding sequences of selenoproteins identified by homology were predicted using the gmhmmp_heuristic.pl program of the Genemark suite (Borodovsky et al. 2003), by considering the best gene model (option -b) obtained after the replacement of the TGA codon with NNN. The Genemark prediction of the *C. jejuni* SelD gene was corrected based on the evidence of a rare CUG start codon (Shaw et al. 2012).

The search of proteins without homology to known selenoproteins was based on previously described methods (Kryukov and Gladyshev 2004). Briefly, open reading frames (ORFs) greater than 90 nt containing potential UGA codons were translated from complete ε-proteobacteria genomes with the Getorf program of the EMBOSS package using a custom genetic code assuming UGA→U decoding. The ORFs were searched for homology using tBLASTn in the same genome set, the results were parsed to count the occurrences of U:C, U:U, and U:X in sequence alignments.

Sequences encompassing 100 nt upstream and downstream candidate SECIS elements identified by the covariance model were translated in full (including stop codons) using the EMBOSS transeq program and assuming UGA→U decoding. This procedure allowed us to observe in tBLASTn alignments U residues immediately followed by a stop codon, as in the case of DUF466 proteins; the same result could be obtained with ORF translations using the tfastx36 program with the “-m BB” option to simulate the BLAST output.

### Identification of Sec tRNAs

The search for tRNA^Sec^ genes was performed using tRNAscan-SE using the option for the maximum sensitivity and the “infernal” model of prokaryotic tRNA^Sec^. The new infernal model (PSELCinf-c.cm) was found to be much more effective in the gene identification than the previous covariance model (PSELC.cm). The final results (see supplementary table S1, Supplementary Material online) were obtained with a search conducted with a specific covariance model for ε-proteobacteria tRNA^Sec^.

### RNA Secondary Structure and Covariance Analysis

The covariance model for bacterial tRNAs (TRNAinf-bact-c.cm) of the Infernal package (Nawrocki and Eddy 2013) was used to align and fold non-Sec tRNAs in the standard cloverleaf structure. The alignment of ε-proteobacterial Sec tRNAs was based on the prokaryotic covariance model of Sec tRNAs (PSELCinf-c.cm). Different secondary structures proposed for Sec tRNAs (see supplementary fig. S1, Supplementary Material online) were evaluated by covariance analysis and by comparing the structure components of the scores obtained by the ε-proteobacteria Sec tRNAs with covariance models assuming 7/5, 8/5, or 9/4 bp in the aminoacyl- and T-stems.

The RNA secondary structure of sequences encoding selenoproteins was analyzed with the Centroid_alifold program (Hamada et al. 2009) using sequence alignments of the region around the TGA codon (−10; +60) of the formate dehydrogenase (Fdh), SelD, and SelW protein families. The prediction of the SECIS structures in individual sequences was based on the bSECISearch program (Zhang and Gladyshev 2005) accessed through the web interface (http://genomics.unl.edu/bSECISearch/). The identification of SECIS elements in the ε-proteobacteria genome set was based on the Cmsearch program (Nawrocki and Eddy 2013) with the covariance model derived from the alignment of figure 3*A* using a cut-off score of 16. The matches of the SECIS elements with the covariance model were assessed by bit scores (*T*) and significance (*P*) as provided by Cmsearch.

Covariance analysis and the drawing of RNA structures were based on the R2R program (Weinberg and Breaker 2011). The instructions used to represent Sec tRNAs in the cloverleaf structure with R2R are given in supplementary figure S2, Supplementary Material online.

### Synonymous–Nonsynonymous Substitutions

The ratio of nonsynonymous substitutions per nonsynonymous site (d*N*) to synonymous substitutions per synonymous site (d*S*) in pairwise comparisons was calculated on the multiple alignment of SelA coding sequences with the maximum-likelihood method implemented in the Codeml program of the PAML package (Yang 2007). Variations of d*N*/d*S* ratios in SelA coding sequences along the ε-proteobacteria tree were analyzed with Codeml by allowing the d*N*/d*S* ratio to vary among particular branches of the tree. Parameters were estimated under models of a single or multiple d*N*/d*S* ratios (up to four) and under models of persistent and episodic rate variations. The likelihoods of the different models were compared using the likelihood ratio test.

### Sequence Alignment and Structure Analysis

Multiple sequence alignments were built with the ClustalW program (Larkin et al. 2007). The presence or absence of the tRNA^Sec^ binding domain in SelA proteins was assessed through comparison with a Hidden Markov Model (Eddy 1996) of the domain. The alignment was analyzed with Genedoc, and the online resource Espript (Robert and Gouet 2014) was used to generate alignment figures. Models of the SelA structure were downloaded from PDB and analyzed with PyMol (http://www.pymol.org). The list of residues involved in protein–protein interactions was downloaded from PDBsum (http://www.ebi.ac.uk/pdbsum). Analysis of residue conservation mapped onto the AaSelA structure was conducted with the Consurf web server (http://consurf.tau.ac.il/) using multiple alignments of SelA sequences from Helicobacteriaceae species possessing or not the Sec-decoding trait.

## Results

### Sec tRNA Genes (*selC*) in ε-Proteobacteria

**Fig. 1.—:**
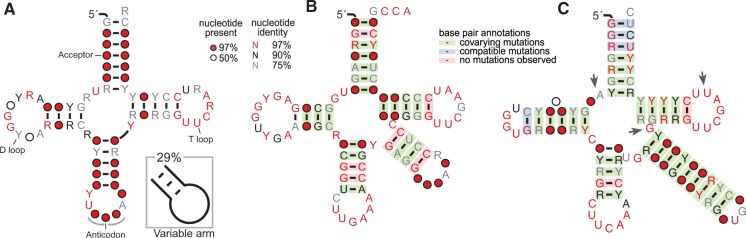
Secondary structure comparison of Sec tRNAs and other tRNAs in ε-proteobacteria. (*A*) Consensus structure of ε-proteobacteria tRNAs (*n* = 1,502); the variable arm found in minor fraction of tRNAs is shown as skeleton diagram in the inset. (*B*) Covariance structure of tRNA^Ser(TGA)^ (*n* = 38), the standard tRNA family more closely related to tRNA^Sec^. (*C*) Covariance structure of tRNA^Sec^ (*n* = 21), characterized by the longer D arm, the extra-long variable arm, and nucleotide positions (indicated by arrows) differing from the consensus. Nucleotide conservation and base pair support from covariance analysis (Weinberg and Breaker 2011) are denoted by colors as shown in the legend.

tRNA genes are usually identified with high accuracy (>99%) in genomic sequences by dedicated programs. An exception is caused by Sec tRNAs, which differ from the tRNA model for the longer D and variable arms and nucleotide positions deviating from the consensus (fig. 1). The general tRNA-finding program tRNAscan-SE (Lowe and Eddy 1997) can optionally identify Sec tRNAs using special covariance models, whereas the eukaryotic tRNA-finding program Pol3scan (Percudani et al. 1997) runs by default a dedicated procedure for their identification; the application of this procedure to the first yeast genome provided evidence for the absence of tRNA^Sec^ in *Saccharomyces cerevisiae*.

Seven tRNA^Sec^ genes were annotated in our ε-proteobacterial genome set (supplementary table S1, Supplementary Material online), whereas a search using tRNAscan-SE with the default parameters identified tRNA^Sec^ genes only in *C. jejuni*, *Campylobacter hominis*, and *C. lari*. However, a high sensitivity search with the Infernal prokaryotic Sec (PSec) model (Nawrocki and Eddy 2013) was able to identify 21 tRNA^Sec^ genes in eight different genera of ε-proteobacteria. Bacterial tRNA^Sec^ were demonstrated to possess an unusually long acceptor stem of 8 bp (Burkard and Söll 1988). Accordingly, the PSec model assumes 8 bp in the aminoacyl stem and 5 bp in the T stem, a structure referred to as the 8/5 structure in contrast to the normal 7/5 structure observed in tRNAs. On the other hand, eukaryotic tRNA^Sec^ are considered to adopt the 9/4 structure (Lukashenko 2010). Comparison of the covariance support for the different models provided evidence for a 7/5 structure for the ε-proteobacteria tRNA^Sec^, similarly to non-Sec tRNAs, but at variance with the structure generally observed in bacteria (fig. 1 and supplementary fig. S1, Supplementary Material online).

An ε-proteobacteria-specific covariance model was built based on a tRNA^Sec^ sequence alignment assuming the 7/5 structure (supplementary fig. S2, Supplementary Material online). A search with the models identified the same 21 tRNA^Sec^ genes with higher score and significance (supplementary table S1, Supplementary Material online) but no additional *selC* candidates. All the identified genes folded well in the tRNA^Sec^ secondary structure (supplementary fig. S3, Supplementary Material online).

### Identification of Annotated and Unannotated Selenoproteins

A search in the complete proteomes of the ε-proteobacteria selected for the analysis revealed ten proteins containing an Sec residue (U): 6 Fdh, 2 selenophosphate synthase (SelD), 1 selenoprotein W (SelW), and 1 thioredoxin (Tr). By searching nucleotide sequences for tBLASTn homology with the specialized trace element database (dbTEU) (Zhang and Gladyshev 2010), we found 40 additional genes potentially encoding Sec residues (supplementary table S2, Supplementary Material online). Most of these sequences corresponded to 5′- or 3′-truncated sequences in GenBank. However, some of the identified genes, particularly those encoding SelW proteins, were completely missed in the genome annotation (supplementary table S2, Supplementary Material online).

Other genes identified by homology with the Sec protein database were found to contain a cysteine instead of an Sec at the same alignment position (supplementary table S3, Supplementary Material online). The possibility of the presence of unknown selenoproteins in the ε-proteobacterial genomes was also considered. For each genome in our set, all possible ORFs longer than 30 amino acids were translated assuming TGA→U decoding and searched with tBLASTn. The results were parsed by calculating the occurrence of U:U and U:C matches with respect to U:X matches in sequence alignments. Using this procedure we rediscovered all the previously identified sequences and one additional candidate selenoprotein: A TGA-containing ORF of *Campylobacter curvus* in which the TGA codon corresponded to cysteines in homologous sequences of *Arcobacter* species (supplementary fig. S4, Supplementary Material online). Although the *C. curvus* protein has significant similarity with thiol peroxidases in dbTEU (best hit: YP_593855.1, *E* = 2 × 10^−^^8^), database sequences contain Sec at a different position (supplementary fig. S5, Supplementary Material online), thus explaining why this protein was not identified as a selenoprotein in our previous search.

### SECIS Elements in ε-Proteobacteria Genes Encoding Selenoproteins

A comparative analysis of RNA secondary structures (Hamada et al. 2009) of the region around the Sec codon indicated the presence of structured RNAs (supplementary fig. S6, Supplementary Material online), and the bSECISearch program (Zhang and Gladyshev 2005) identified the presence of optimal SECIS elements in all sequences except in a putative SelW protein of *Campylobacter fetus* (see supplementary table S2, Supplementary Material online). Interestingly, an optimal SECIS element was also identified downstream the TGA codon of the *C. curvus* peroxidase, providing further evidence for its identification as a selenoprotein (supplementary fig. S4, Supplementary Material online).

**Fig. 2.—:**
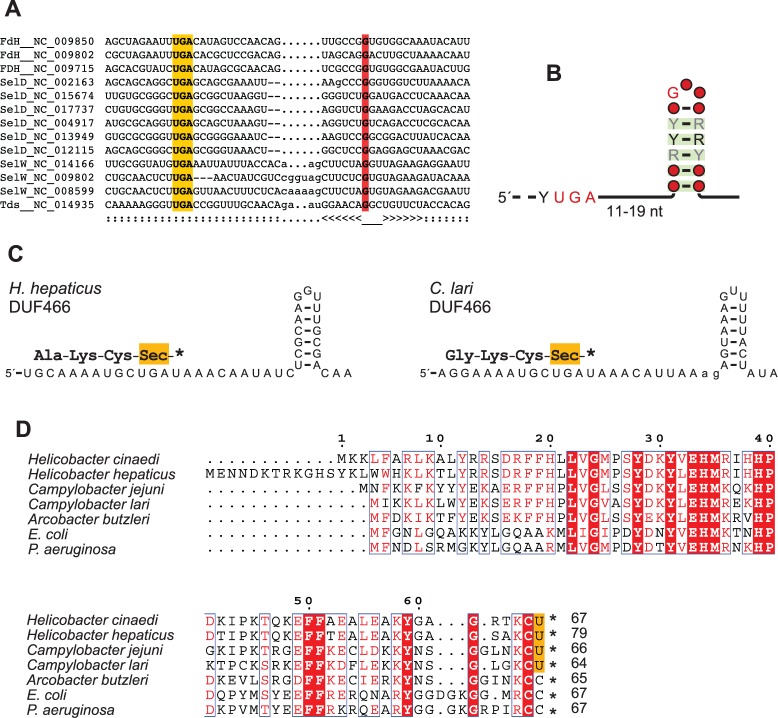
SECIS elements and identification of novel selenoproteins in ε-proteobacteria. (*A*) Nucleotide alignment of the region around the Sec codon in RNAs encoding known selenoproteins in ε-proteobacteria. The proposed base pairing is represented below the alignment in Stockholm notation; the TGA Sec codon is shaded in orange; the invariant guanosine in the hairpin loop is shaded in red. (*B*) Depiction of the consensus structure of SECIS elements in the full set of ε-proteobacterial selenoproteins; color code from covariance analysis is as in figure 1. (*C*) Proposed secondary structure of the identified SECIS elements of the *H. hepaticus* and *C. lari* DUF466 proteins. Protein translation is shown above the RNA sequence. (*D*) Multiple alignments of the DUF466 protein family in various bacteria. Residues conserved in all sequences are shaded in red; the putative Sec residues are shaded in orange.

From the output of the bSECISsearch program and the comparative analysis of secondary structures, the presence of a common 5/6-bp hairpin—known as the “apical loop” (Chen et al. 1993; Zhang and Gladyshev 2005)—was evident in all the analyzed sequences a few nucleotides downstream the TGA codon. This structure bears close resemblance to the “minimal” SECIS element required for the in vivo Sec incorporation in *Escherichia coli *Fdh (Liu et al. 1998). A structure-guided alignment of sequences representative of the various selenoprotein families was used to build a covariance model of this common SECIS element in ε-proteobacteria (fig. 2*A*). The covariance analysis of the full set of Sec-encoding sequences revealed strong support for base pairs 1–5 of the hairpin stem and confirmed the presence of an invariant guanosine (Zhang and Gladyshev 2005) in the hairpin loop (fig. 2*B*). The model was able to correctly identify the SECIS region in the full sequence set with significant bit scores (*T* > 16; *P* < 10^−^^5^). Although the model did not include homologs to the thiol peroxidase of *C. curvus*, this sequence received a significant score (*T* = 18.7; *P* = 1.4 × 10^−^^6^) when searched with the model.

### Identification of DUF466 as a Selenoprotein in Campylobacterales

The SECIS covariance model was utilized for a search in the ε-proteobacteria genome set obtaining 131 additional candidates beside those already identified. Homology searches conducted with the full translations of the frame including the putative TGA codons revealed the presence of SECIS elements downstream of sequences encoding proteins of the DUF466 (Domain of Unknown Function) family (fig. 2*C*). In particular, SECIS elements with significant scores were identified downstream of the TGA codon of DUF466 proteins of *Helicobacter hepaticus* (*T* = 19.6; *P* = 8 × 10^−^^7^), *Helicobacter cinaedi* (*T* = 19.8; *P* = 7 × 10^−^^7^), and *C. lari* (*T* = 11.1; *P* = 9 × 10^−^^5^). The presence of optimal SECIS elements in these sequences was confirmed by bSECISearch analysis. The putative Sec residue encoded by DUF466 is preceded by a cysteine and followed by a TAA/TAG stop codon; in homologous proteins, the equivalent position is occupied by a cysteine preceded by another cysteine and followed by a stop codon (fig. 2*D*). The fact that Sec is the very last residue of the sequence explains why this protein was not identified by our previous search, as the tBLASTn alignments did not include terminal “U” residues.

There is no similarity between the DUF466 proteins and known selenoproteins, as assessed by hidden Markov model searches (hmmsearch; *E* > 1), and there are no other identified proteins possessing a carboxyl-terminal Sec.

### Evolution of the Sel Machinery in ε-Proteobacteria

**Fig. 3.—:**
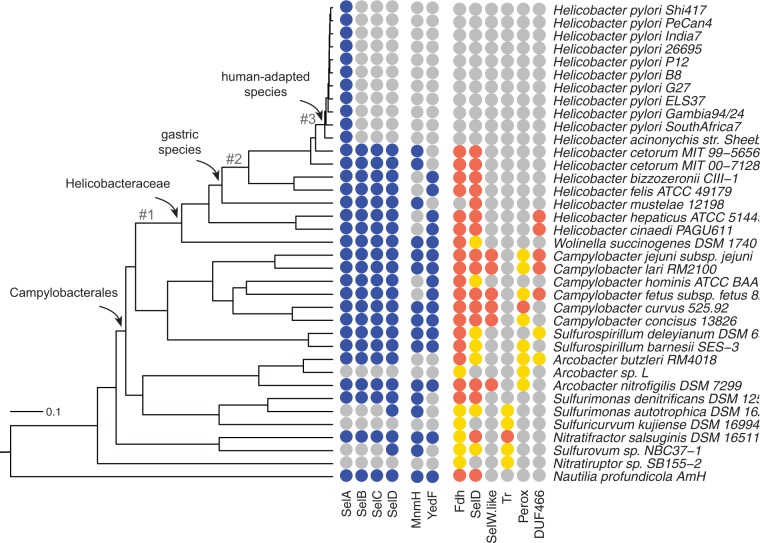
Distribution of SelA and related proteins in ε-proteobacteria genomes. The ultrametric tree represents a whole-genome phylogeny of ε-proteobacteria. Tree branches relevant for the evolutionary analysis of the *selA* gene are numbered. The presence or absence of genes is shown alongside the tree. A colored circle indicates a gene identified in the bacterium genome; a gray circle indicates a gene not identified in the bacterium genome. In selenoproteins, a red circle represents a protein with Sec, whereas a gold circle represents a protein with cysteine at the same alignment position. Note that SelD is both a component of the Sel machinery and a selenoprotein.

To analyze the distribution of Sel proteins in the context of ε-proteobacteria evolution, we considered species in which a highly resolved whole-genome phylogeny is available (Wang and Wu 2013). After exclusion of very similar genomes (Genomic Similarity Score > 0.95) (Moreno-Hagelsieb et al. 2013), the species phylogram was converted into a chronogram by applying a molecular clock model (Paradis 2013). The resulting ultrametric tree (fig. 3) highlights the deep divergence of Campylobacterales (Battistuzzi et al. 2004), and the very recent divergence of *H. pylori* and *Helicobacter acinonychis* (large feline pathogen) which is thought to have occurred less than 0.4 Ma through host jump from early human populations (Eppinger et al. 2006). For each genome in the ε-proteobacteria phylogeny, we determined the presence of the genes involved in Sec protein incorporation, genes involved in selenium metabolism, and genes encoding selenoproteins (fig. 3). The *selC* tRNA^Sec^ genes and genes encoding selenoproteins were identified by the dedicated search procedures described above, whereas *SelA*, *SelB*, and *SelD* were identified by homology with reference proteins. Most genes were identified at the protein level, although a search with translated genomic sequences was also performed, which allowed us to identify a missing SelD protein in *C. curvus*, and to correct the coding sequence boundaries of SelA in *Helicobacter cetorum*, *Helicobacter felis*, and *Helicobacter bizzozeronii* (supplementary table S1 and fig. S7, Supplementary Material online). Homology searches were also used for the identifications of two genes that are often found in ε-proteobacteria and other organisms near *sel* genes: *mnmH*, encoding the enzyme tRNA 2-selenouridine synthase involved in wobble base modification of specific anticodons (Wolfe et al. 2004), and *yedF*, encoding a SirA-like selenium metabolism protein of undefined function (Zhang et al. 2008).

As evident from the gene distribution in figure 3, a complete Sel system for the incorporation of Sec into proteins is found in most species and was presumably present in the common ancestor of ε-proteobacteria. Because of the metabolic relation existing among Sel proteins, the distribution of SelA, SelB, SelC, and SelD follows a predictable scheme (Romero et al. 2005; Zhang et al. 2006): They are either present or absent together or, alternatively, only SelD is present, meaning that selenophosphate is used for purposes other than Sec biosynthesis. The presence of MnmH and YedF implies the presence of SelD, probably for the requirement of selenophosphate, but not necessarily of the other Sel components (Zhang et al. 2008). The predicted scheme is respected in the ε-proteobacteria tree except for the descendants of the recent common ancestor of *H. pylori* and *H. acinonychis*.

### SelA Is Encoded by a Functional Gene in *H. pylori–H. acinonychis*

The gene distribution reported in figure 3 shows that, in general, the presence of SelA in a genome implies the presence of the other genes for Sec incorporation and one or more genes encoding selenoproteins. The exception is represented by *H. pylori* and *H. acinonychis*. To the best of our efforts, we could not identify other *sel* genes nor genes encoding selenoproteins in these two species. The possibility was considered that SelA is a nonfunctional remnant (i.e., a pseudogene) of an ancestral gene involved in Sec biosynthesis. However, this hypothesis is challenged by the lack of frameshift or missense mutations in the SelA sequences of *H. acinonychis* and the numerous-sequenced strains of *H. pylori*.

We analyzed the ratio of nonsynonymous to synonymous substitutions (d*N*/d*S*) of the *selA* genes encoded by these two species and found values substantially lower than 1 in all the pairwise comparisons (supplementary fig. S8, Supplementary Material online). The d*N*/d*S* value of SelA (0.33 ± 0.1) is higher than the average d*N*/d*S* value (0.14 ± 0.07) reported for 786 core genes of *H. pylori* (Didelot et al. 2013), indicating weak purifying selection. However, it is comparable to the values observed for core metabolic genes such as protoporphyrinogen oxidase (HP0381; d*N*/d*S* = 0.32), shikimate kinase (HP1249; d*N*/d*S* = 0.34), and thymidylate kinase (HP1474; d*N*/d*S* = 0.37). Overall, this analysis provides a strong evidence that SelA is still a functional gene in the species that have lost the other components of the Sel system.

### Accelerated Evolution of SelA in Gastric *Helicobacter* Species

The topology of the tree constructed with the SelA protein sequences can be reconciled with the organism tree with minor local rearrangements (supplementary fig. S9, Supplementary Material online), consistent with vertical inheritance of the *selA* gene in ε-proteobacteria. However, disproportionately long branches are observed in the gene tree after the common ancestor of gastric *Helicobacter* (compare branches #2 and #3 in fig. 3 and supplementary fig. S9, Supplementary Material online), suggesting accelerated evolution in this lineage.

Changes in the selection pressure acting on *selA* genes were inferred by estimating the variation of d*N*/d*S* ratios along the branches of the ε-proteobacteria tree (table 1). A two-rate model of d*N*/d*S* was found to be significantly better than a single-rate model; and among the two-rate models, the best likelihood was obtained by assuming a variation of d*N*/d*S* in the branch leading to the common ancestor of *H. pylori* and *H. acinonychis* (branch #3 in fig. 3). A better fit was however obtained with a three-rate model suggesting a first increase of d*N*/d*S* after the split of *Helicobacter mustelae* from the other gastric species (branch #2 in fig. 3). A four-rate model provided only a slightly better fit and suggested a decrease rather than an increase of d*N*/d*S* in the lineage leading to Helicobacteriaceae (branch #1 in fig. 3). In all cases analyzed, persistent changes of d*N*/d*S* (i.e., the variation is propagated to descending branches) provided a better fit than episodic changes (i.e., the variation is not propagated to descending branches), consistent with a long-term variation of the selection pressure (supplementary fig. S10, Supplementary Material online).

**Table 1:** Parameter Estimates under Models of Variable d*N*/d*S* Ratios of the *selA* Gene among ε-Proteobacteria Lineages

Model	d*N*/d*S* on Branches				Log Likelihood	LRT
	#0	#1	#2	#3		
H0: (1 rate)	0.11	=#0	=#0	=#0	−23,198.31	
H1: (2 rates)	0.05	=#0	=#0	0.23	−23,126.97	3.5E-33^a^
H2: (3 rates)	0.02	=#0	0.08	0.24	−23,111.25	1.1E-08^b^
H3: (4 rates)	0.02	0.003	0.08	0.24	−23,106.83	0.002^c^

Note.—Branches are numbered as in figure 3; #0 represents the tree root.

^a^H1 versus H0, df = 1.

^b^H2 versus H1, df = 1.

^c^H3 versus H2; df = 1.

### Specific Truncation of the SelA N-Terminal Domain in *H. pylori and H. acinonychis*

Annotated SelA proteins of *H. acinonychis* (HaSelA) and *H. pylori* (HpSelA) are shorter than the typical SelA proteins as they lack a region of about 50 amino acids at the N terminus. At variance with apparently truncated SelA of other *Helicobacter* species that could be extended by examination of the genomic sequences (see supplementary fig. S7, Supplementary Material online), there is evidence of a closed frame upstream of HaSelA and HpSelA (supplementary fig. S11, Supplementary Material online). The missing region is not part of the PLP-dependent domain (fold-type I) of SelA, which appears to be complete in the two species. The analysis of the region upstream SelA in *H. pylori* also revealed the presence of a short ORF not annotated in the reference genome of the strain 26695 (Tomb et al. 1997). This ORF, encoding a short basic polypeptide (K + R = 35%; pI = 11.9) without similarity to known proteins, is interposed between HP1512 (NikR-regulated outer membrane protein) and HP1513 (SelA) (supplementary fig. S11*A*, Supplementary Material online). Although the ORF is annotated only in few genomes, it was found by tBLASTn searches at the same locus in all *H. pylori* strains as well as in *H. cetorum* and *H. acinonychis*. Sequence comparison at the protein and nucleotide level provides evidence that the ORF is coding (supplementary fig. S11*B* and *C*, Supplementary Material online). There is no sequence relationship between the short basic protein upstream SelA and the N-terminal domain missing in HaSelA and HpSelA.

### Conserved and Nonconserved Features of HpSelA

SelA is responsible for the synthesis of Sec directly on its tRNA, which has previously been loaded with serine. The 3D structure of SelA has been recently solved for the enzyme from *Aquifex aeolicus* (Itoh et al. 2013). The protein is organized as a homodecamer with a pentagonal ring structure composed of five SelA dimers which collectively bind ten tRNA^Sec^ molecules. Each tRNA is cooperatively bound by two dimers that form the tRNA binding pocket at the interaction surface between each other, whereas the active site is located at the interface between the subunits of each dimer (Itoh et al. 2013).

**Fig. 4.—:**
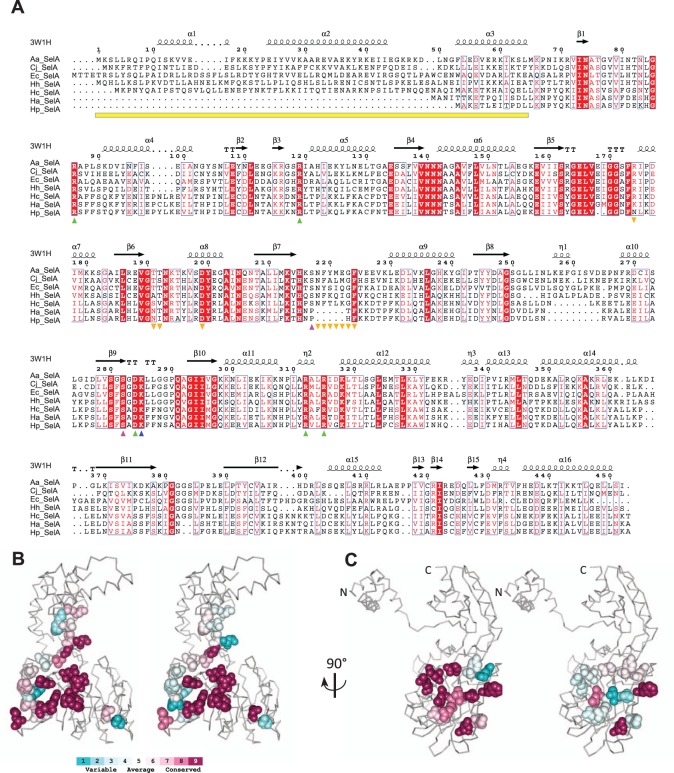
Conserved and nonconserved features in HpSelA. (*A*) Multiple alignment of SelA sequences from *A. aeolicus* (AaSelA), *E. coli* (EcSelA), *C. jejuni* (CjSelA), *H. hepaticus* (HhSelA), *H. cetorum* (HcSelA), *H. acinonychis* (HaSelA), and *H. pylori* 26695 (HpSelA). Secondary structure elements inferred from the crystal structure of AaSelA (PDB: 3W1H) are shown above the alignment. The yellow bar indicates the tRNA^Sec^ binding domain. Downwards orange triangles indicate dimer–dimer interacting regions. The upwards blue triangle indicates the PLP-binding Lys; upwards purple triangle indicates residues interacting with PLP. Upwards green triangles indicate selenophosphate-binding residues. (*B*) Consurf analysis (Ashkenazy et al. 2010) of residues involved in hydrogen-bonded intradimer and (*C*) interdimer interactions in Helicobacteriaceae species with (left panels) or without (right panels) the Sec decoding trait.

The alignment of SelA sequences from *H. pylori* and *H. acinonychis* with SelA sequences from other ε-proteobacteria, *E. coli*, and *A. aeolicus* (fig. 4), shows that the N-terminal sequence missing in HpSelA and HaSelA corresponds to the domain that binds tRNA^Sec^ in the AaSelA structure (particularly the D-arm and T-loop). This N-terminal domain is present in all Sec-utilizing organisms, including *H. cetorum* (*H. pylori* and *H. acinonychis* closest relative). The absence of this domain in the two non-Sec-utilizing organisms suggests that HpSelA and HaSelA lost their capability of binding tRNA^Sec^ along with the loss of the other *sel* genes. The analysis of residue conservation mapped onto the protein structure (Ashkenazy et al. 2010) and the analysis of d*N*/d*S* variation among sites show that SelA residues involved in intradimer interactions have similar conservation profiles among Helicobacteriaceae (fig. 4*B* and supplementary table S4, Supplementary Material online). This is at variance with residues involved in dimer–dimer interaction, which are poorly conserved in species lacking the Sec-decoding trait (fig. 4*C* and supplementary table S5, Supplementary Material online). In particular, a region located between residues 218 and 224 of AaSelA, reported to be crucial for dimer–dimer interaction (Itoh et al. 2014), is almost completely absent in HpSelA and HaSelA (see fig. 4*A*). Furthermore, Itoh et al. (2014) found that the quadruple mutation Tyr220Pro-Asp199Arg-Thr191Tyr-Thr192Tyr abolishes pentamerization and yields a dimeric SelA. Of these four residues, only Asp199 is conserved in both HaSelA and HpSelA, whereas Tyr220 is located in the deleted dimer–dimer interaction region, Thr191 is substituted with Asn in HpSelA and HaSelA and Thr192 is substituted with Ile in HpSelA. Similarly, Arg174, involved in a hydrogen bond with Asp199 of a neighboring dimer in AaSelA, is not conserved in *H. acinonychis* and *H. pylori*.

In contrast, the catalytically active residues appear to be conserved in HpSelA and HaSelA (see fig. 4). The Lys285 of AaSelA that covalently binds PLP is strictly conserved in both HpSelA (all strains) and HaSelA and so are Arg86, Arg312 and Arg315 that bind selenophosphate. Arg86 is also supposed to be involved in the catalytic mechanism by helping substrate protonation, hinting that an enzymatic activity is retained by HpSelA and HaSelA. Also conserved are the Arg119 and Asp284 residues involved in the correct positioning of Arg312 and Arg86, respectively. The analysis of the available PDB structures identifies two residues able to form hydrogen bonds with PLP, namely Ser217, which could form an H bond engaging the 3′ hydroxyl group of PLP, and Ser282, which could contact one of the oxygen atoms belonging to the phosphate group of PLP. Although Ser282 is conserved in all sequences, Ser217 is substituted with Pro in *H. pylor*i and *H. acinonychis*. Finally, it is worth noting that dimeric AaSelA is not catalytically active, as the tRNA^Sec^ is bound by two dimers and processed by the active site of the neighboring dimers (Itoh et al. 2013); however, direct binding of a smaller substrate directly to the SelA active site would eliminate the need of the pentagonal ring structure for catalysis.

## Discussion

Prompted by the presence of a putative *selA* gene in *H. pylori* and the lack of information on selenium and Sec metabolism in this bacterium, we have carried out a comparative analysis of the Sec-decoding trait and the selenoproteome in Helicobacteriaceae and related ε-proteobacteria. Our analysis confirms that selenoproteins and components of the Sec-decoding trait require special attention in genome annotation. Most of the selenoproteins and tRNA^Sec^ genes identified in this work were misannotated or unannotated in sequence databases (see supplementary tables S1 and S2, Supplementary Material online).

tRNA^Sec^ genes were largely missed by searching with the standard tRNA model, but accurately identified by searching with the specific infernal PSec model. Nevertheless, the tRNA^Sec^ structure in ε-proteobacteria shows some differences with respect to the PSec model, as these tRNAs appear to form 7 bp instead of 8 in the aminoacyl stem. The length of the aminoacyl stem in tRNA^Sec^ is relevant to the positioning of the tRNA-bound amino acid at the SelA active site (Itoh et al. 2013) and has been shown to affect SelB binding in *E. coli* (Baron and Böck 1991).

Known selenoproteins could be identified through comparisons with the specialized dbTEU database. However, the use of a covariance model of SECIS elements and a variation in the BLAST search procedure enabled the identification of an additional selenoprotein family (DUF466), representing the first example of a carboxyl-terminal Sec. As the local algorithm of BLAST does not extend the alignment to include terminal mismatches (e.g., U:C), proteins ending with an Sec can escape identification by the standard search methods. A more exhaustive search in the available genomes could reveal the presence of other protein families possessing a terminal Sec. Proteins belonging to DUF466 are found in more than 1,200 species of Proteobacteria, Actinobacteria, and Firmicutes (Finn et al. 2014). They are typically short proteins of about 70 amino acids possessing an invariant cysteine at the C terminus. Although there is no functional information on this domain, the identification of family members with an Sec suggests a redox activity (Fomenko et al. 2007) for the C-terminal residue of DUF466. The configuration of a conserved cysteine followed by an Sec (see fig. 2*D*) is found in animal thioredoxin reductase, in which the two neighbor residues form a selenylsulfide bond that acts as a redox center for the reduction of the thioredoxin disulfide bond (Cheng et al. 2009).

The results presented in this work provide strong evidence that *H. pylori* does not incorporate Sec into proteins nor use selenium for tRNA wobble bases modifications. The *selA* gene of *H. pylori* originates from a component of the Sec-decoding trait that has been recruited for a different function after loss of the trait during Helicobacteriaceae evolution.

### SelA Modifications Associated with the Loss of the Sec-Decoding Trait

**Fig. 5.—:**
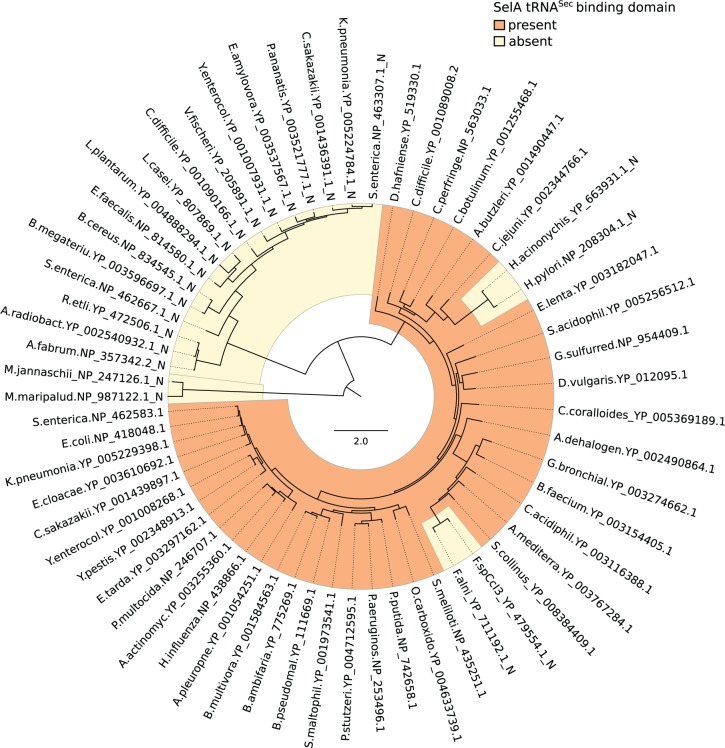
Distribution of the tRNA^Sec^ binding domain in prokaryotic SelA proteins. Midpoint-rooted circular phylogram representing a maximum-likelihood phylogeny of SelA proteins in prokaryotes. The presence or absence of the N-terminal tRNA-binding domain is highlighted with different colors. Labels are aligned to tips and indicate the abbreviated taxon name followed by the sequence accession numbers.

A change of the selection pressure acting on the *selA* gene was revealed by variations of d*N*/d*S* ratio in gastric *Helicobacter* species (see table 1). The gene appeared to be under strong selection pressure in most ε-proteobacteria lineages and in the common ancestor of Helicobacteraceae (d*N*/d*S* = 0.02). The selection pressure changed first in an early ancestor of gastric *Helicobacter* (d*N*/d*S* = 0.08) and then, more recently, in the lineage leading to *H. acinonychis* and *H. pylori* (d*N*/d*S* = 0.24). Given that the analysis of amino acidic substitutions suggests loss of the decameric organization of SelA in these two species, this latter change of d*N*/d*S* is consistent with relaxation of the selection pressure in sites previously involved in the quaternary structure. Loss of quaternary interactions is a classical case for accelerated evolution due to relaxed selection pressure (Horuk et al. 1980). We do not have an explanation for the variation of the d*N*/d*S* ratio occurred in the early evolution of gastric *Helicobacter*, although we note that in these species the *selA* gene has lost the genetic linkage with other component of the Sel system, at variance with what is observed in other Helicobacteriaceae (supplementary fig. S12, Supplementary Material online). The loss of the Sec-decoding trait in *H. pylori*–*H. acinonychis* has been accompanied by sequence and structure modifications in the SelA protein. Among these, the most evident is the loss of the N-terminal tRNA^Sec^ binding domain. As the absence of this domain can be the hallmark of SelA homologs not involved in Sec incorporation, a relevant question is whether truncated SelA proteins are present in other species. A search for the N-terminal domain in the full prokaryotic phylogeny of SelA proteins (fig. 5) reveals the presence of an entire SelA lineage lacking the tRNA^Sec^ binding domain. Members of this early diverged SelA subfamily are found in bacterial species lacking the Sec-decoding trait (e.g., *Agrobacterium radiobacter*) or species having the trait and a full-length SelA paralog (e.g., *Salmonella enterica*). A similar situation is found in Archaea, particularly in Methanococci. Along with Eukaryotes, Archaea use a different mechanism and a different gene (*O*-phosphoseryl-tRNA:selenocysteinyl-tRNA synthase) for Sec biosynthesis. Besides the archaeal Sec synthetase gene, Methanococci (e.g., *Methanocaldococcus jannaschii*) also possess SelA homologs apparently lacking affinity for tRNA^Sec^ (Kaiser et al. 2005). In general, proteins belonging to the main SelA lineage conserve the N-terminal domain and are found in organisms possessing the Sec-decoding trait. Loss of the N-terminal domain in this lineage occurred independently in *Helicobacter* and in some *Frankia* spp. (e.g., *F. alni* and *F. CcpI3*). As in *H. pylori*, the other components of the Sel system are absent in these bacteria.

### Biological Role of SelA Proteins Not Involved in Sec-Decoding

Different hypotheses can explain the observed pattern for the presence and the absence of the tRNA^Sec^ domain in SelA proteins. A possibility is that SelA proteins are in general endowed with an alternative function that can take over as the main function when the Sec-decoding trait is lost or when there is a paralogous protein involved in the trait. Alternatively, SelA proteins could have independently evolved different functions upon dismissal of the Sec-decoding trait. Both hypotheses are tenable, as PLP-dependent enzymes can display “catalytic promiscuity” (Han et al. 2001), and are extremely prone to functional divergence: More than 150 different enzymatic activities are currently listed in the PLP-dependent superfamily (fold-type I) to which SelA belongs (Percudani and Peracchi 2009). Even though the available evidence does not allow one to guess a precise function for SelA homologs of *H. pylori* and other species, bioinformatics analysis and biochemical reasoning can infer some general features of those proteins. The presence of the PLP cofactor suggests an enzymatic activity on a compound containing a primary amine (typically amino acids and derivatives), whereas the conservation of residues binding the phosphate moiety of SePO_3_ suggests involvement of a phosphorylated substrate. Finally, although the binding to tRNA^Sec^ can be excluded, the possibility remains for the interaction with a substrate containing an RNA moiety.

### When Was the Sec-Decoding Trait Lost in the *H. pylori* Lineage?

Our analysis provides evidence that the loss of the Sel system occurred before the separation of *H. pylori* and *H. acinonychis*, but after the common ancestor of *H. pylori* and *H. cetorum* (see fig. 3). It has been estimated that *H. pylori* and *H. acinonychis* diverged from a common ancestor already adapted to the human host about 200,000 years ago (Eppinger et al. 2006). In our ultrametric tree, this value corresponds to about 450,000 years for the divergence of *H. pylori* and *H. cetorum*, suggesting that the loss of the Sec-decoding trait becomes fixed in the *H. pylori* lineage in a relatively narrow time scale (less than 300,000 years). This observation, together with the evidence of other independent events of loss or gain of *sel* genes and selenoproteins in the ε-proteobacteria phylogeny (see fig. 3), highlights the remarkable evolutionary plasticity of the Sel system (Zhang et al. 2006).

### Why Has the Sec-Decoding Trait Been Lost in *H. pylori?*

It has been observed that the presence of an Sec-encoding Fdh is the main determinant for the maintenance of an Sec-decoding trait (Romero et al. 2005). The absence of Sec-decoding in *H. pylori* could thus be a natural consequence of the loss of Fdh and lack of a selection pressure for the maintenance of the trait. Noticeably, Fdh was independently lost in *H. mustelae*, a species that has instead maintained the trait and an Sec-encoding SelD as the sole selenoprotein (see fig. 3). SelD can function with a cysteine at the active site, so the substitution of this residue in *H. mustelae* would render the other components of the system dispensable. As the loss of the Sec-decoding trait temporally coincided with adaptation of a *H. pylori* progenitor to the human host, the possibility should be considered that loss of the trait has an adaptive value for the bacterium and its interaction with the host. A relation has been reported between the presence of the trait and preference for an anaerobic lifestyle (Zhang et al. 2006). In this regard, *H. pylori* appears to be more oxygen tolerant than other *Helicobacter* and related genera. *Wolinella succinogenes*, the closest *Helicobacter* relative, requires less than 2% O_2_ for growth, *H. hepaticus* and *Helicobacter bilis* can tolerate up to 10% O_2_, whereas *H. pylori* can grow in particular conditions even at the atmospheric (21%) oxygen pressure (Bury-Moné et al. 2006). Finally, it is possible that the loss of the Sec-decoding trait in this exquisitely adapted human inhabitant had beneficial consequences for the host, by avoiding competition for an essential element and selenium deficiency in infected individuals. This hypothesis could be verified by comparing the gastric and circulating selenium levels of animals infected by *Helicobacter* species possessing or not the Sec-decoding trait.

## Supplementary Material

Supplementary figures S1–S12 and tables S1–S5 are available at *Genome Biology and Evolution *online (http://www.gbe.oxfordjournals.org/).

Supplementary Data
