# Supplementary material for: Evolution of the Selenoproteome in Helicobacter pylori and Epsilonproteobacteria
Source: Genome Biol Evol. 2015 Sep 4;7(9):2692–704. doi: 10.1093/gbe/evv177 (PMC4607533; doi:10.1093/gbe/evv177)
Supplement: Supplementary Data [file supp_7_9_2692__index.html]

Evolution of the Selenoproteome in Helicobacter pylori and Epsilonproteobacteria — Supplementary Data 

# Evolution of the Selenoproteome in *Helicobacter pylori* and Epsilonproteobacteria

## Supplementary Data

files

- Supplementary Data - pdf file
