# Supplementary material for: Evolution of the Selenoproteome in Helicobacter pylori and Epsilonproteobacteria
Source: Genome Biol Evol. 2015 Sep 4;7(9):2692–704. doi: 10.1093/gbe/evv177 (PMC4607533; doi:10.1093/gbe/evv177)
Supplement: Supplementary Data [file supp_evv177_Figures_Tables_supplementary_SelA.pdf]

Evolution of the selenoproteome in Helicobacter pylori and Epsilonproteobacteria

Cravedi, P., Mori, G., Fischer, F., Percudani, R.

Supplemntary figures and tables

Figure S1. **Evidence for a 7/5 secondary structure in ε–proteobacteria $tRNA^{Sec}$**

Figure S2**. Structural alignment of representative ε–proteobacteria $tRNA^{Sec}$**

Figure S3. **Secondary structures of $tRNA^{Sec}$ identified in ε–proteobacteria**

Figure S4. **Evidence for a selenocysteine thiol peroxidase (Prx) in *C. curvus***

Figure S5. **Comparison of *C. curvus* thiol peroxidase with homologous selenoproteins**

Figure S6. **Predicted RNA secondary structures around Sec-encoding codons of ε–proteobacteria**

Figure S7**. Redefinition of coding sequence boundaries in SelA sequences**

Figure S8. **dN/dS ratios in the SelA coding sequences of *H. pylori* an *H. acinonychis***

Figure S9. **Comparison of the organism tree and the SelA tree in ε–proteobacteria**

Figure S10**. Comparison of different models of dN/dS variation of the *selA* gene along branches**

Figure S11. **Evidence for a closed frame and presence of a coding ORF upstream of *H. pylori* SelA**

Figure S12. **Genetic neighborhood of the *selA* gene in Helicobacteriaceae**

Table S1**. Genes involved in the Sec-deconding trait and selenometabolism identified in Epsilonproteobacteria**

Table S2. **Sec-encoding proteins identified in Epsilonproteobacteria genomes**

Table S3**. Position of Sec-encoding codons and equivalent Cys-encoding codons in ϵ-proteobacteria genomes**

Table S4**. Conservation of SelA residues involved in hydrogen-bonded intra-dimer interactions**

Table S5**. Conservation of SelA residues involved in hydrogen-bonded dimer-dimer interactions**

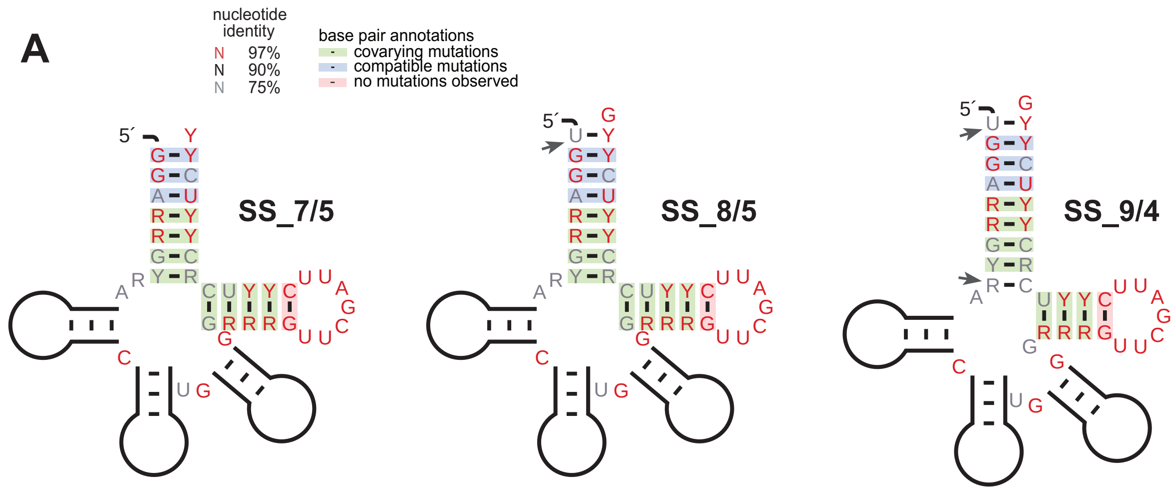

B

	SS_7/5	SS_8/5	SS_9/4
NC_017761.1:683686-683781	**27.58**	26.94	25.67
NC_014935.1:1763108-1763204	**27.43**	26.94	25.49
NC_012115.1:1239494-1239590	**35.37**	34.82	33.22
NC_008599.1:335720-335815	**35.85**	35.36	33.92
NC_009802.1:1788312-1788407	**27.29**	26.13	24.92
NC_002163.1:1432373-1432468	**29.22**	28.74	27.63
NC_013949.1:727834-727929	26.80	**27.76**	26.93
NC_004917.1:419603-419698	33.91	**34.48**	32.98
NC_009714.1:280399-280494	**28.05**	27.57	26.60
NC_012039.1:343444-343539	**19.75**	19.28	18.32
NC_017735.1:1339955-1340050	**29.70**	29.21	28.50
NC_013512.1:2018206-2018302	**21.97**	20.81	19.53
NC_014166.1:977634-977731	**24.27**	23.78	22.36
NC_018002.1:2149993-2150089	**21.97**	20.81	19.53
NC_017737.1:723764-723859	**29.70**	29.21	28.50
NC_009850.1:1529457-1529553	**24.96**	24.47	22.87
NC_015674.1:696633-696729	**32.52**	31.88	30.40
NC_009715.1:1748632-1748727	**30.98**	29.82	28.62
NC_014810.2:420555-420651	33.16	**33.73**	32.48
NC_007575.1:862218-862314	**22.37**	21.88	20.24
NC_005090.1:797104-797199	**22.00**	21.52	20.78

Figure S1. **Evidence for a 7/5 secondary structure in ε–proteobacteria tRNA^{Sec}**. (A) Consensus secondary structures of ε–proteobacteria tRNA^{Sec} showing lack of covariance support for bp. num. 1 in the 8/5 model and for bp. num. 1 and num. 9 in the 9/4 model (arrows). (B) Secondary structure (SS) components of the bit scores obtained for 21 ε–roteobacteria tRNA^{Sec} with three different covariance models using the Cmsearch program (Nawrocki and Eddy 2013). Best scores for each tRNAs are shown in bold. The three covariance models were based on artificial sequences generated by the Cmemit program from an initial tRNA^{Sec} model. Each models had the same HMM component, as obtained by building a model without SS information (cmbuild --ignorant), and different SS components, as obtained by building complete models of alignments comprising 7/5, 8/5, and 9/4 base pairs in the aminoacyl and T stems. The SS components of Cmsearch scores were calculated by subtracting the HMM score to the scores obtained with the complete models.

```
# STOCKHOLM 1.0
#=GF AU    Infernal 1.0.2

lcl|NC_012115.1:1239495-1239589        GGAGGGGAUGUGaU.CC.UGGUGGACACCCCGGGCUUCAAACCCGGU.GGGGCGGCAGGUgACUGUCUGCccGGAAGGUUCGAUUCCUUCCCCCUUCC
lcl|NC_002163.1:1432374-1432467        GGAAGAUUAGCGuA.UC.UGGUGAUCGCCACUGACUUCAAAUCAGAU.GAAAGGAUAGUU.GACUAUUCUUUGGGGAGUUCGAUUCUCUCAUCUUCUC
lcl|NC_004917.1:419604-419697          GGAGAGCAAACGuC.UC.UGGUGGGCGUCUUGGGCUUCAAACCCAUU.GAAGGAUUAGGU.GUCUAAUUCUUGGGGAGUUCGAUUCUCUCGCUCUCCU
lcl|NC_009714.1:280400-280493          GGAGAGUAAGUC.G.UGcCGGUGGCGAUCGCGGACUUCAAAUCCGAU.GAAAGGGCGGUU.GACCGUUCUUUGGGGGGUUCGAUUCCCUCACUCUCUC
lcl|NC_009850.1:1529458-1529552        GGGAGGUUAGUG.UaCC.UGGUGGGCACCACAGGCUUCAACCCUGAUuGACGGUUUUGAU.GACAACGCCGUGGGAGGUUCGAUUCCUUCACCUUCUC
lcl|NC_012039.1:343445-343538          GGAAGGUUAGCUuA.UC.UGGUGAUGGCCACUGACUUCAAAUCAGAU.GAAAGGGUAGUU.GACUACUUUUUGGGGAGUUCGAUUCUCUCACCUUCUC
lcl|NC_014166.1:977635-977730          GGGAGAUAAGUG.UaCC.UGGUGGGCACCACAGGCUUCAACCCUGAUuGACGGUUUAUGUgUAAUACGCCGUGGGAGGUUCGAUUCCUUCAUCUUCUC
lcl|NC_014810.2:420556-420650          GGAGAGCGAGCA.UgGC.UGGUGCGUGCCUUGGGCUUCAAACCCAAA.GAGCGUUUAGGUgUCCUAAGCGUUGCAAAGUUCGAUUCUUUGGCUCUCUU
lcl|NC_017735.1:1339956-1340049        GGAGAGUAAGCG.U.AUcUGGUGUAUGCCUUGGGCUUCAAACCCAUA.GAGUGCCUAGAU.GUCUAGGUGCUGAGAAGUUCGAUUCUUCUACUCUCUC
lcl|NC_017761.1:683687-683780          GGAGGGCAAGCG.U.CUcUGGUGGGCGUCGUGGGCUUCAAACCCAUU.GAAGGGUUAGGU.GUCUAAUCCUUGGGGAGUUCGAUUCUCUCGCCUUCCU
#=GC SS_cons                           (((((((,,<<<.<.<<.____>>>>>>,<<<<<_______>>>>>,.,<<<<<<<<<,,.,,>>>>>>>>>,<<<<<_______>>>>>))))))):
#=GC RF                                [accep]=====.=.[=Dloop=]============acd=======[==========vlp===========]======[Tloop]=====[accep]=
#=GC R2R_LABEL                         ......j..........................................1.....................2.........................3

#=GF R2R set_dir pos0 90 f
#=GF R2R place_explicit 3 3-- 0 1 0 0 0 0

#=GF R2R multistem_junction_bulgey j disable_auto_flip_place_explicit J0/base 0 1.62706 2.29987 0 0 -270 J1/base 0 4.83116
1.01097 0 0 0 J2/base 0 5 -3 0 0 -50 J3/base 0 2.32414 -2.52817 0 0 -90

#=GF R2R ifdef skeleton
#=GF R2R SetDrawingParam scaleMeasurementsBy 0.25
#=GF R2R SetDrawingParam backboneWidth 1.5pt
#=GF R2R SetDrawingParam pairBondWidth 0.5pt
#=GF R2R endif

#=GF Makefile skeleton-with-pairbonds
//
```

Figure S2**. Structural alignment of representative ε–proteobacteria tRNA^{Sec}**. The proposed base pairing is represented below the alignment in Stockholm notation. The structural model assumes 6 base pairs in the D arm (with possible single-base bulges) and 7 base pairs in the aminoacyl arm (without bulges). The R2R instructions used to draw tRNAs in cloverleaf structures (see figs. 1, S1, and S3) are reported below the alignment.

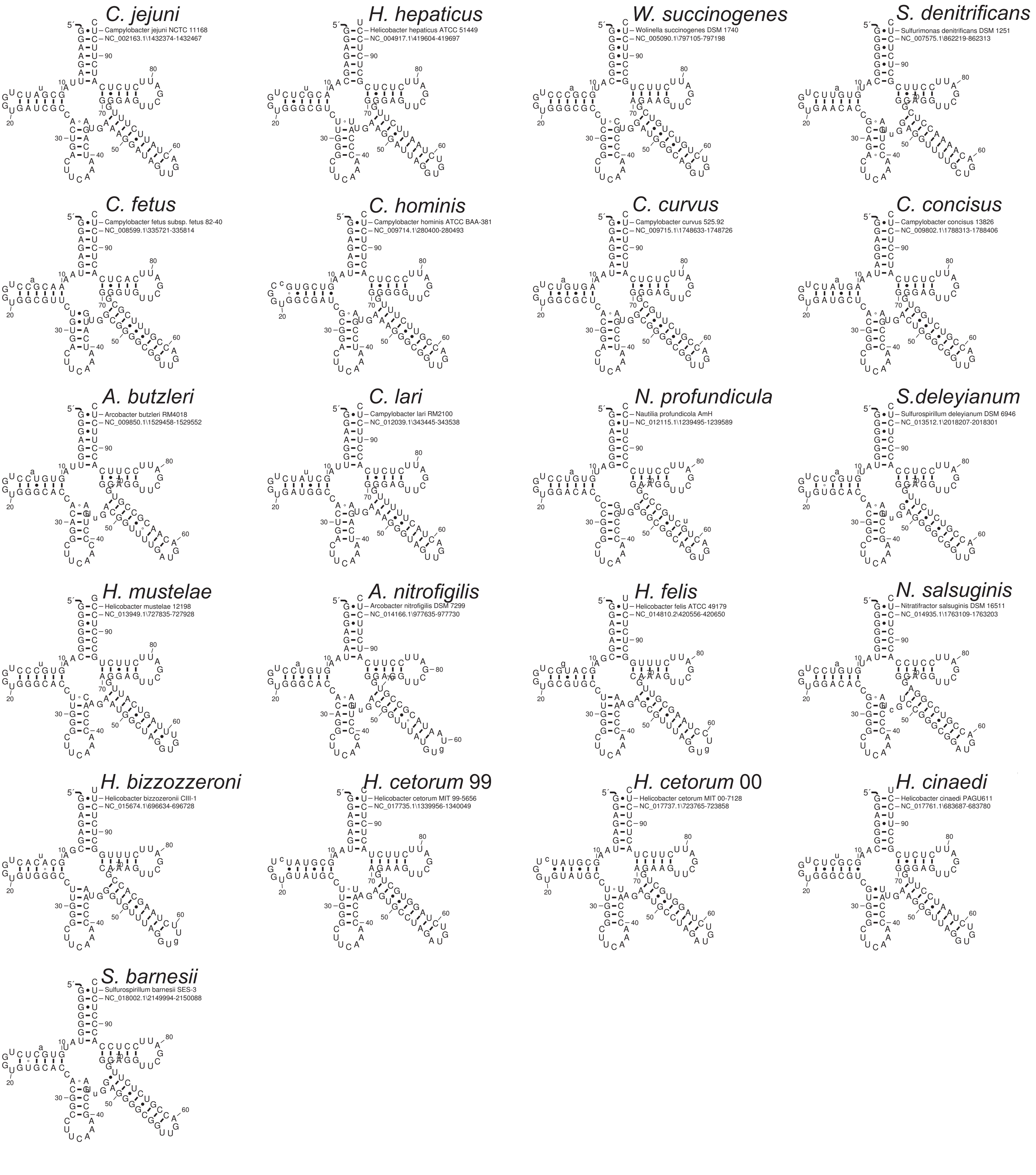

Figure S3. **Secondary structures of tRNA^{Sec} identified in ε–proteobacteria**. Structures were drawn in the standard tRNA layout using the R2R program based on the structural alignment in Stockholm format obtained with the Cmalign program of the Infernal package (Nawrocki & Eddy 2013).

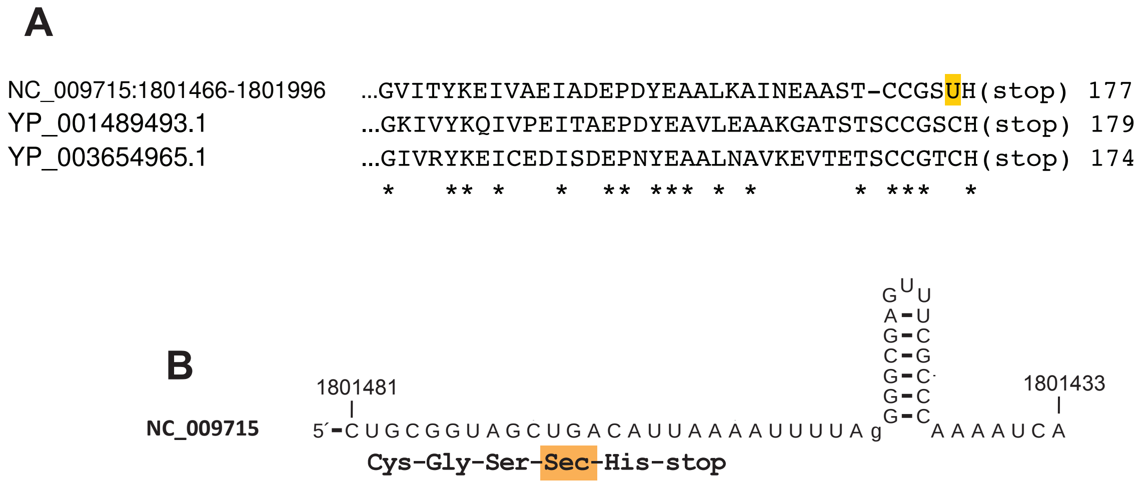

Figure S4. **Evidence for a selenocysteine thiol peroxidase (Prx) in *C. curvus***. (A) The C-terminal region of a TGA-containing open reading frame of *Campylobacter curvu*s (NC_009715:1801466-1801996) is aligned with homologous proteins of *Arcobacter butzleri* (YP_001489493.1) and *Arcobacter nitrofigilis* (YP_003654965.1); the putative selenoecysteine (U) residue is shaded in orange. (B) The secondary structure of the SECIS element of *C. curvu*s Prx as predicted by the bSECISsearch program (Zhang and Gladyshev 2005); the protein translation is shown below the RNA sequence.

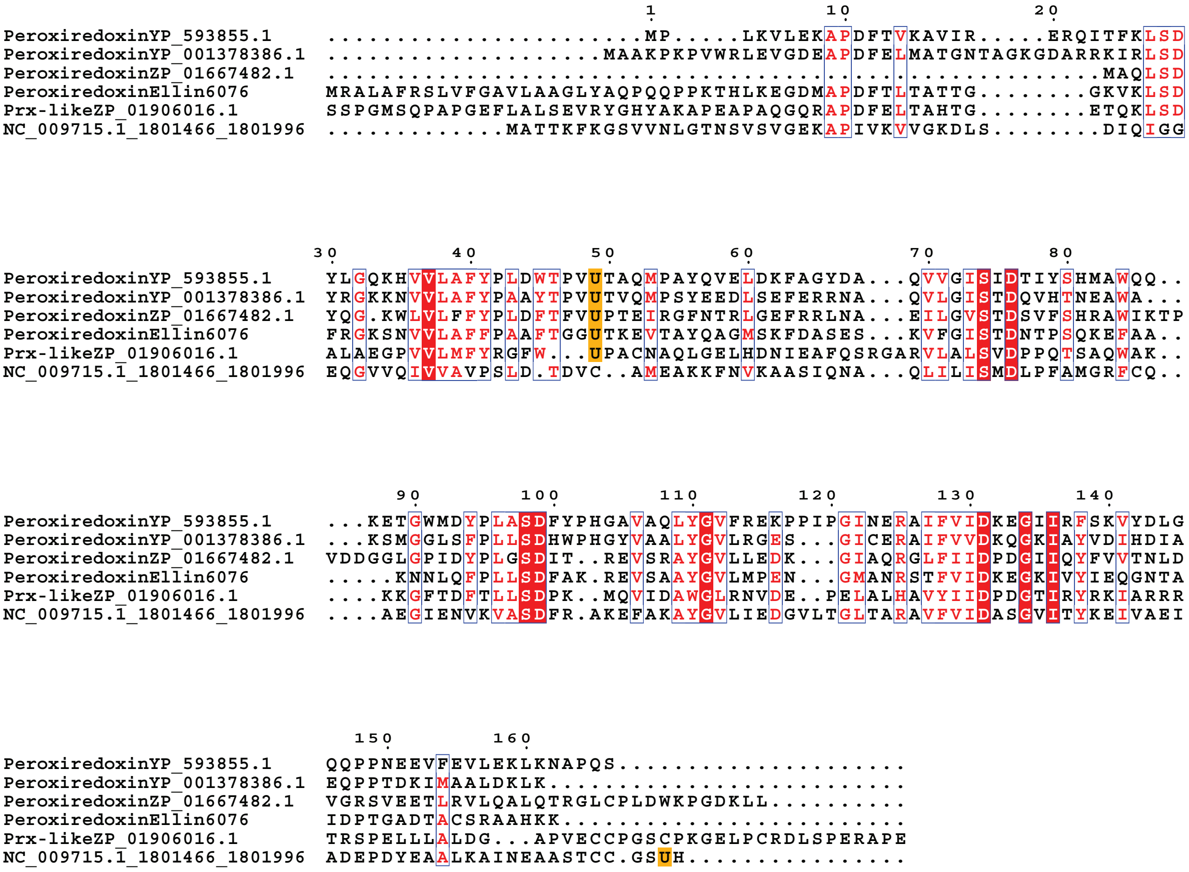

Figure S5. **Comparison of *C. curvus* thiol peroxidase with homologous selenoproteins**. The translation of a putative Sec-containing ORF of *C. curvus* (NC_009715.1_1801466_1801996) is aligned with the five top scoring blastp hit of dbTEU. Conserved columns are shaded in red, putative Sec (U) residues are shaded in orange. The alignment was conducted with Clustalw with manual adjustments.

```
>Fdh
AGCWMGAAUYUGACAYAGYGCMACAGUCGCCGGUGUGGCGAAUACWUKKGGUUAUGGYGCKAUGACAAAYCAY
......................................................................... (g=0.25)
......................................................................... (g=0.5)
.................(((.......((((.....)))).................)))............. (g=1)
...........(.....((((.....(((((.....)))))...............)))).....)....... (g=2)
........((((.((..((((.....(((((.....)))))...............))))..)).))))).... (g=4)
.......(((((.((.(((((.....(((((.....)))))...............))))).)).)))))... (g=6)
.......(((((.((((((((.....((((((...))))))...............)))))))).)))))... (g=8)
.....(.(((((.((((((((..(.(((((((...))))))...)....(....)))))))))).)))))).. (g=16)

>SelD
AKSYGCKGGUUGAGCKGCYAARSURGRYCYGKMRGRYCUWWMMCAAAYYWYYKSCaakCUYAYHYAAMMYCAU
......................................................................... (g=0.25)
.........................((((.....))))................................... (g=0.5)
.........................((((.....))))................................... (g=1)
........................((((((...))))))................................... (g=2)
........................((((((...))))))................................... (g=4)
....((.......)).(((.....((((((...))))))..............))).................. (g=6)
....((.......)).((((....((((((...)))))).............)))).................. (g=8)
....((.......))(((((((..((((((...))))))..........))))))).................. (g=16)

>SelW
YUGCAAYYYUUGA---AAYUWYCKYCCRSWAGCUKCUMGKKUWGMAGAUGARHUAMWAAABRAUUUYMSWGRU
......................................................................... (g=0.125)
......................................................................... (g=0.25)
.................................(((.......)))........................... (g=0.5)
................................(((((.....)))))........................... (g=1)
................................(((((.....)))))........................... (g=2)
................................((((((...))))))........................... (g=4)
...........((...(...............((((((...))))))................)))...... (g=6)
......(((((((...(.(((........)))((((((...))))))................))))))))). (g=8)
.....((((((((...(((((((....)))))((((((...)))))).................)))))))))) (g=16)
```

Figure S6. **Predicted RNA secondary structures around Sec-encoding codons of ε–proteobacteria selenoproteins**. The analysis was conducted with the Centroid_alifold program based on multiple alignments of the Fdh, SelD, and SelW families using different weights (g) for base pairs. The Sec-encoding UGA codon and the common hairpin structure are highlighted in yellow.

A

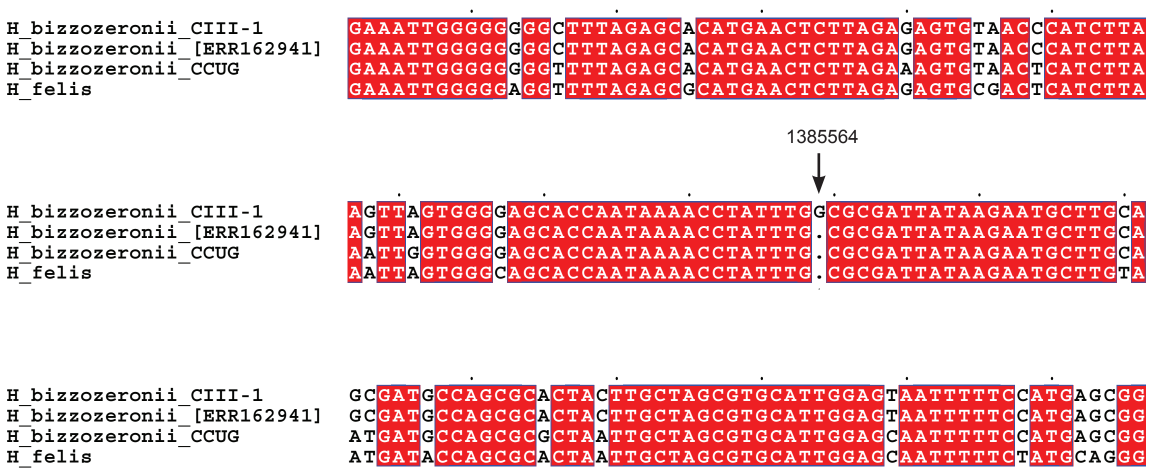

B

Genome Accession	Species	Annotated CDS	Predicted CDS	Sequence variation
NC_014810.2	*H. felis* ATCC 49179	703832..704323	702989..704323	
NC_015674.1	*H. bizzozeronii* CIII-1	1384976..1385575<br>1385634..1386278	1384976..1386278	g.1385564del
NC_017735.1	*H. cetorum* MIT 99-5656	944261..945457	944093..945457	
NC_017737.1	*H. cetorum* MIT 00-7128	c264740..265936	c264740..266104	

Figure S7**. Redefinition of coding sequence boundaries in SelA sequences.** (A) Portion of the SelA coding sequence of the *H. bizzozeronii* CIII-1 genome (NC_015674.1:1384976..1386278) aligned with homologous sequences from *H. felis* and other *H. bizzozeronii* isolates ('CCUG' and 'ERR162941'). The possible frameshift error in the *H. bizzozeronii* CIII-1 SelA sequence is indicated by an arrow. The sequence labeled ' ERR162941' was obtained by a Velvet assembly (k-mer=31; cov_cutoff=auto) of *H. bizzozeronii* reads available in the NCBI Sequence Reads Archive (Project accession: ERX138816; run: ERR162941). (B) Comparison of the annotated SelA coding sequence boundaries with the coordinates inferred by tblastn searches. The sequence variation in NC_015674.1 is proposed based on the above alignment and the evidence for a Sec-decoding trait in this organism (see fig. 3).

	H.acino-nychis	H.p 26695	H.p Shi417	H.p Gam-bia94/2	H.p P12	H.p G27	H.p B8	H.p ELS37	H.p India7	H.p PeCan4
**H. acynonychis**										
**H.p_26695**	0.261									
**H.p_Shi417**	0.289	0.365								
**H.p_Gambia94/2**	0.265	0.446	0.369							
**H.p_P12**	0.235	0.316	0.276	0.473						
**H.p_G27**	0.258	0.327	0.291	0.598	0.371					
**H.p_B8**	0.235	0.309	0.284	0.441	0.230	0.325				
**H.p_ELS37**	0.249	0.385	0.340	0.521	0.411	0.460	0.348			
**H.p_India7**	0.229	0.244	0.294	0.495	0.262	0.298	0.220	0.459		
**H.p_PeCan4**	0.257	0.536	0.282	0.377	0.247	0.327	0.324	0.357	0.293	
**H.p_SouthAfrica**	0.343	0.295	0.248	0.375	0.288	0.311	0.296	0.342	0.246	0.261

Figure S8. **dN/dS ratios in the SelA coding sequences of *H. pylori* an *H. acinonychis***. The ratio of non-synonymous substitutions per non-synonymous site (dN) to synonymous substitutions per synonymous site (dS) in pairwise comparisons was calculated on the multiple alignment of SelA coding sequence with the maximum-likelihood method implemented in the Codeml program of the PAML package.

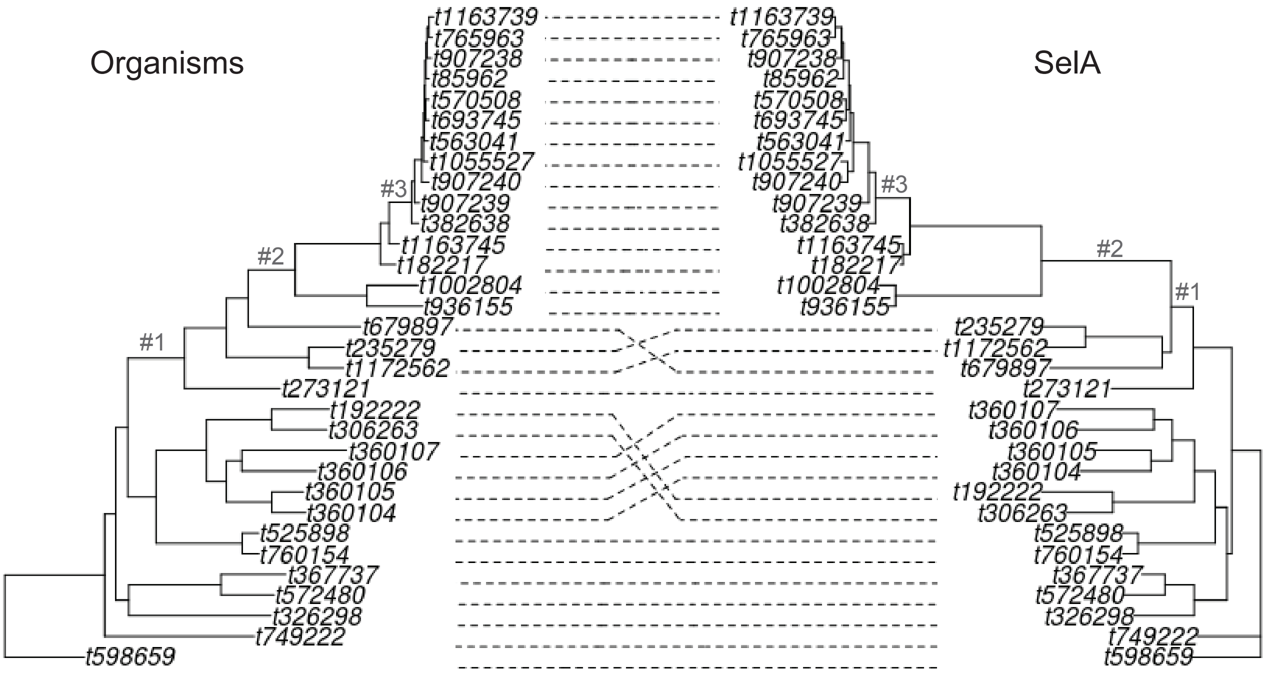

Figure S9. **Comparison of the organism tree and the SelA tree in ε–proteobacteria**. The left tree represents the maximum-likelihood phylogeny obtained with 454 proteins of ε–protebacteria (Wang and Wu 2013), the right tree represents the maximum-likelihood phylogeny of SelA proteins. Dashed lines connect tips with the same taxonomy id. Crossing dashed lines represent the rearrangements required to reconcile the two trees, as determined by the Cophyplot routine of the APE package.

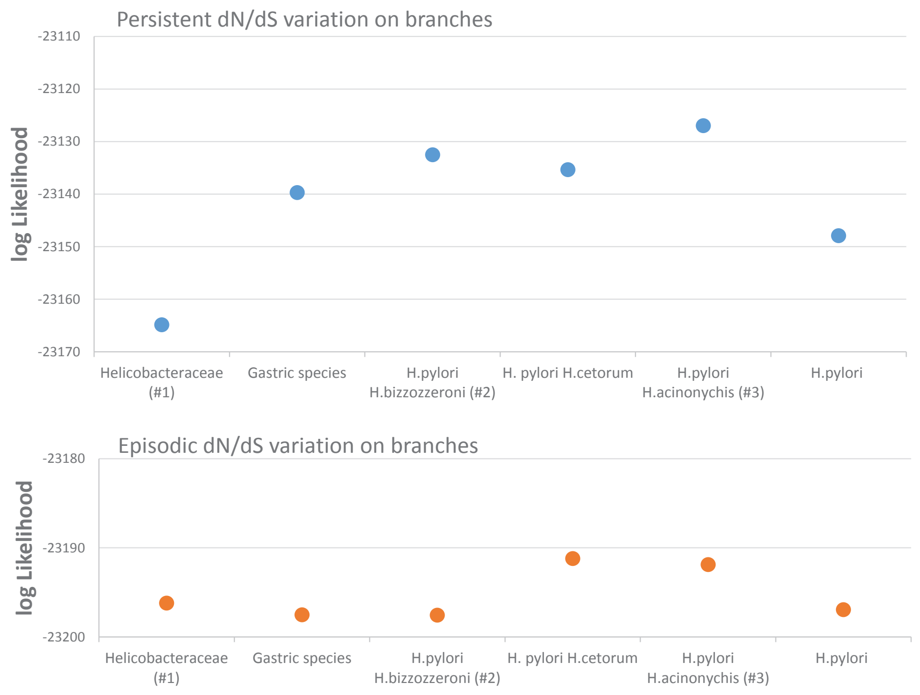

Figure S10**. Comparison of different models of dN/dS variation of the *selA* gene along branches**. The log Likelihood of models assuming variation of dN/dS in the *selA* gene along the Helicobacteraceae phylogeny is plotted for different branches of the tree. Tree topology and branch numbering is as in figs. 3 and S9. Upper panel: variations of dN/dS were propagated to the descending branches (persistent variations). Lower panel: variations of dN/dS were not propagated to the descending branches (episodic variations).

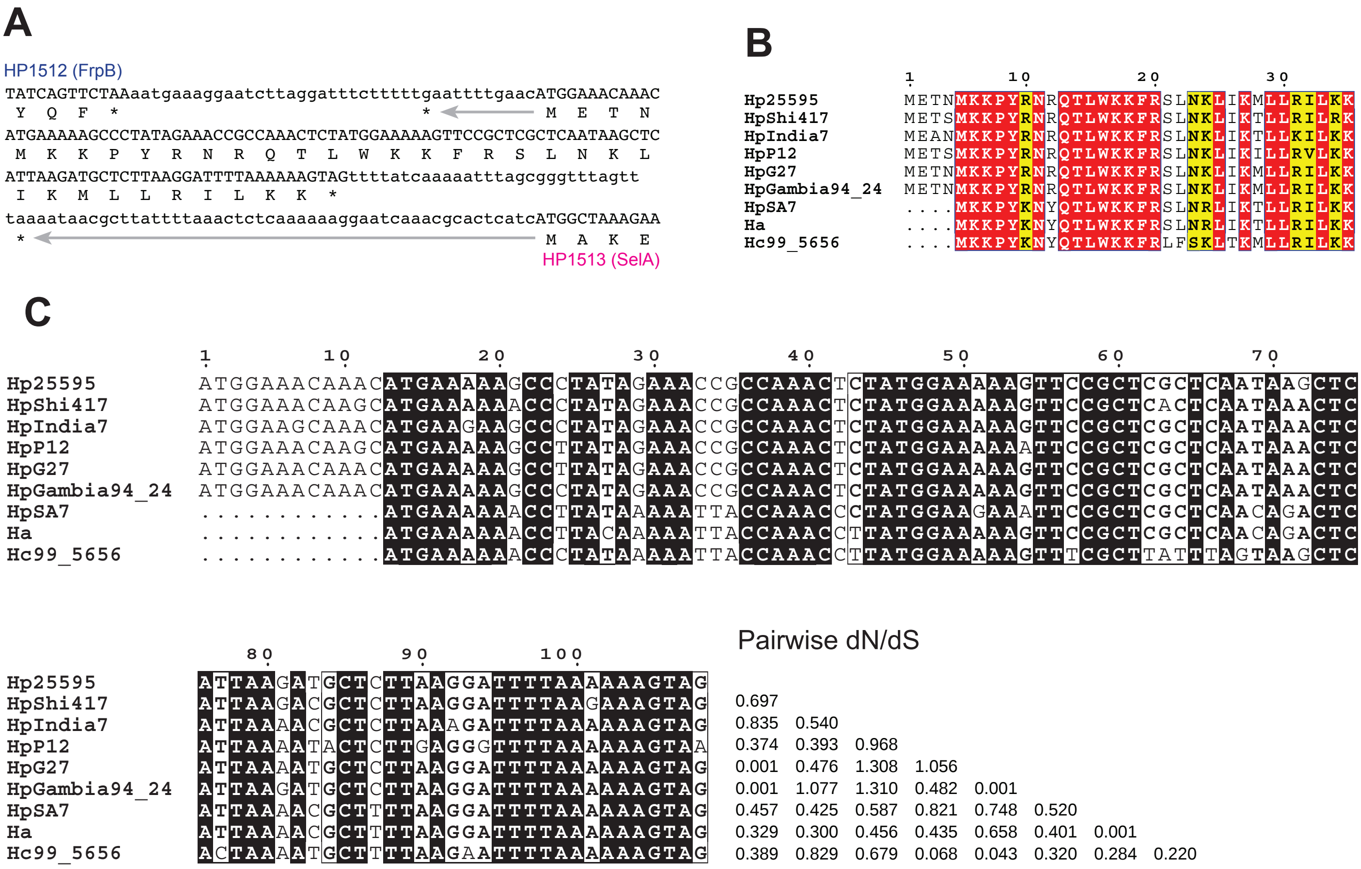

Figure S11. **Evidence for a closed frame and presence of a coding ORF upstream of *H. pylori* SelA.** (A) DNA sequence of the intergenic region between HP1512 (NikR-regulated outer membrane protein) and HP1513 (SelA). Proposed coding sequences are shown in capital letters; translation is shown below the sequence. In-frame downstream and upstream STOP codons are represented by asterisks. (B) Protein alignment of the SelA 5'-ORF in Helicobater genomes, highlighting strictly conserved positions (red) and conservative amino acid substitutions (yellow). (C) Coding sequence alignment of the SelA 5'-ORF along with dN/dS ratios from pairwise comparisons.

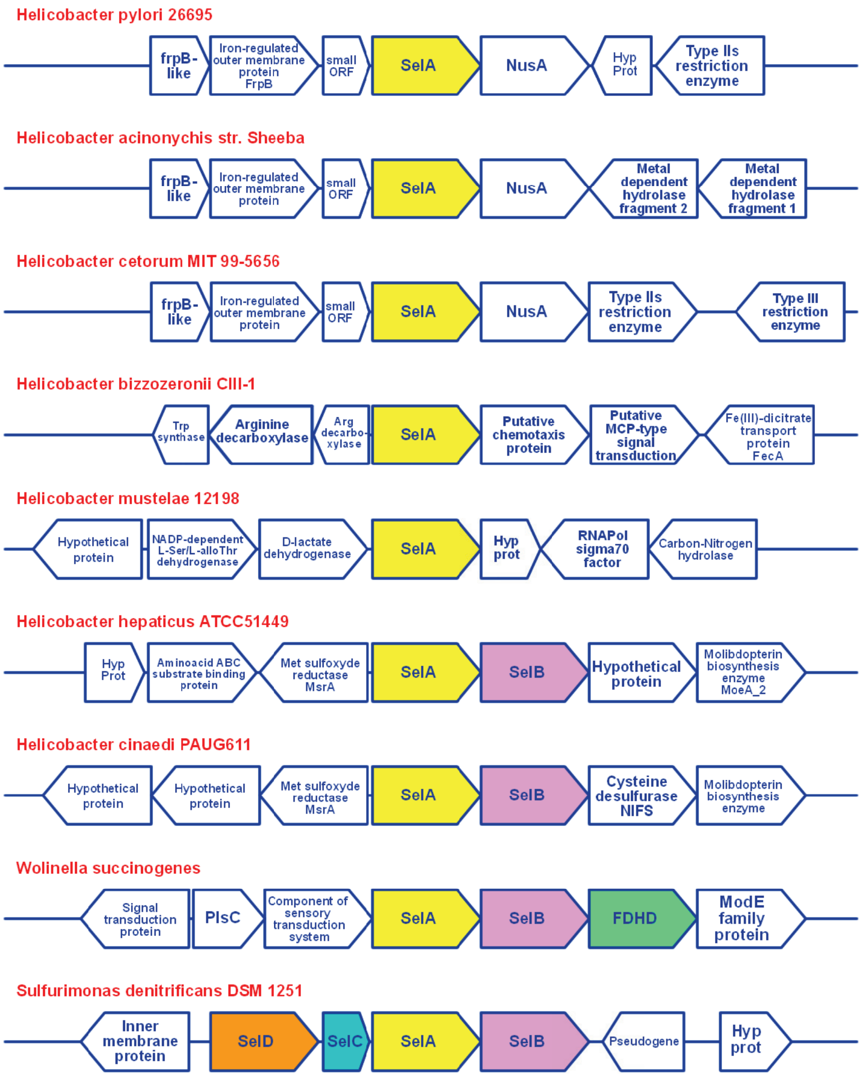

Figure S12. **Genetic neighborhood of the *selA* gene in Helicobacteriaceae**. Boxes representing genes involved in Sec-decoding traits and genes encoding selenoproteins are colored. Same-colored boxes represent orthologous genes across the genomes.

Table S1. Genes involved in the Sec-deconding trait and selenometabolism identified in Epsilonproteobacteria [a]

Taxid	Species	SelA	SelB	SelC	SelD	MnmH	YedF
367737	Arcobacter butzleri RM4018	YP_001490447.1 5e-107	YP_001490448.1 7e-134	NC_009850.1:1529457-1529553 87.47	YP_001490437.1 4e-75		
572480	Arcobacter nitrofigilis DSM 7299	YP_003655158.1 8e-109	YP_003655159.1 1e-134	NC_014166.1:977634-977731 82.23	NC_014166.1:976595-977629 4e-76	YP_003655616.1 5e-96	YP_003655154.1 5e-20
944547	Arcobacter sp. L						
360104	Campylobacter concisus 13826	YP_001466208.1 7e-106	YP_001466209.1 5e-180	NC_009802.1:1788312-1788407 90.36	NC_009802.1:1726587-1727609 1e-89	YP_001466733.1 4e-23	YP_001467547.1 2e-32
360105	Campylobacter curvus 525.92	YP_001408945.1 4e-112	YP_001408946.1 3e-179	NC_009715.1:1748632-1748727 88.40	NC_009715.1:c1656276-1657316 2e-76	YP_001408347.1 2e-24	YP_001408903.1 7e-34
360106	Campylobacter fetus subsp. fetus 82-40	YP_892679.1 2e-122	YP_892680.1 3e-156	NC_008599.1:335720-335815 85.58	NC_008599.1:308394-309425 2e-76		YP_891541.1 2e-33
360107	Campylobacter hominis ATCC BAA-381	YP_001406339.1 8e-103	YP_001406340.1 1e-158	NC_009714.1:280399-280494 92.75	YP_001407288.1 1e-76		YP_001407289.1 1e-34
192222	Campylobacter jejuni ATCC 700819	YP_002344766.1 1 0.0	YP_002344767.1 0.0	NC_002163.1:1432373-1432468 96.65	NC_002163.1:c1439168-1440190 5e-176	YP_002343934.1 2e-26	YP_002344884.1 3e-102
306263	Campylobacter lari RM2100	YP_002575855.1 5e-160	YP_002575856.1 0.0	NC_012039.1:343444-343539 93.39	NC_012039.1:335663-336694 3e-102	YP_002575503.1 7e-29	YP_002574971.1 1e-59
382638	Helicobacter acinonychis str. Sheeba	YP_663931.1 8e-51					
1002804	Helicobacter bizzozeronii CIII-1	NC_015674.1:1384976-1386278 3e-32	YP_004606986.1 6e-118	NC_015674.1:696633-696729 86.98	NC_015674.1:c790126-791136 3e-57		YP_004607782.1 2e-18
182217	Helicobacter cetorum MIT 00-7128	NC_017737:c264740-266104 5e-65	YP_006222572.1 2e-98	NC_017737.1:723764-723859 95.12	NC_017737.1:c1260015-1261040 5e-65	YP_006223140.1 5e-28	
1163745	Helicobacter cetorum MIT 99-5656	NC_017735.1:944093-945457 6e-63	YP_006221630.1 4e-99	NC_017735.1:1339955-1340050 95.12	NC_017735.1:c1168443-1169468 6e-63	YP_006220516.1 3e-29	
1172562	Helicobacter cinaedi PAGU611	YP_006235949.1 4e-96	YP_006235950.1 9e-122	NC_017761.1:683686-683781 97.49	NC_017761.1:c901529-902617 1e-56		YP_006235255.1 3e-24
936155	Helicobacter felis ATCC 49179	NC_014810.2:702989-704323 1e-39	YP_004073919.1 3e-103	NC_014810.2:420555-420651 89.24	NC_014810.2:108644-109654 4e-58		YP_004073732.1 4e-12
235279	Helicobacter hepaticus ATCC 51449	NP_860271.1 4e-92	NP_860272.1 3e-119	NC_004917.1:419603-419698 99.46	NC_004917.1:c1658852-1659898 3e-54		NP_861267.1 1e-25
679897	Helicobacter mustelae 12198	YP_003516820.1 2e-90	YP_003517390.1 7e-104	NC_013949.1:727834-727929 89.16	NC_013949.1:c958499-959524 9e-54	YP_003516376.1 2e-30	
85962	Helicobacter pylori 26695	NP_208304.1 1e-47					
1163739	Helicobacter pylori Shi417	YP_006225087.1 1e-51					
907240	Helicobacter pylori Gambia94/24	YP_005781246.1 1e-47					
570508	Helicobacter pylori P12	YP_002302119.1 2e-49					
563041	Helicobacter pylori G27	YP_002267046.1 3e-49					
693745	Helicobacter pylori B8	YP_003728036.1 4e-47					
1055527	Helicobacter pylori ELS37	YP_005425646.1 3e-48					
907238	Helicobacter pylori India7	YP_005782778.1 7e-52					
765963	Helicobacter pylori PeCan4	YP_003927743.1 4e-52					
907239	Helicobacter pylori SouthAfrica7	YP_005772001.1 1e-50					
598659	Nautilia profundicola AmH	YP_002607717.1 1e-104	YP_002607718.1 2e-146	NC_012115.1:1239494-1239590 87.95	YP_002607680.1 3e-82	YP_002607706.1 6e-23	YP_002607685.1 1e-28
749222	Nitratifractor salsuginis DSM 16511	YP_004168822.1 1e-122	YP_004168821.1 1e-134	NC_014935.1:1763108-1763204 87.12	YP_004167329.1 8e-77	YP_004167330.1 1e-92	YP_004168824.1 3e-27
387092	Nitratiruptor sp. SB155-2						
709032	Sulfuricurvum kujiense DSM 16994						
563040	Sulfurimonas autotrophica DSM 16294				YP_003891308.1 6e-51	YP_003891307.1 1 0.0	
326298	Sulfurimonas denitrificans DSM 1251	YP_393345.1 2e-113	YP_393346.1 4e-135	NC_007575.1:862218-862314 80.85	NC_007575.1:861177-862211 4e-75	YP_393674.1 2e-105	
760154	Sulfurospirillum barnesii SES-3	YP_006405073.1 4e-105	YP_006405074.1 4e-142	NC_018002.1:2149993-2150089 81.95	YP_006405044.1 3e-75	YP_006404163.1 5e-30	YP_006405045.1 2e-36
525898	Sulfurospirillum deleyianum DSM 6946	YP_003305129.1 1e-105	YP_003305130.1 5e-141	NC_013512.1:2018206-2018302 81.95	YP_003305100.1 6e-76	YP_003304359.1 3e-26	YP_003305101.1 3e-33
387093	Sulfurovum sp. NBC37-1				YP_001359133.1 1e-54	YP_001359132.1 1e-105	
273121	Wolinella succinogenes DSM 1740	NP_907051.1 1e-101	NP_907050.1 3e-132	NC_005090.1:797104-797199 81.79	NP_907200.1 2e-71	NP_908105.1 1e-31	NP_907203.1 3e-29

[a]Protein identification was based on homology searches with bona fide SelA (YP_001001036.1), SelB (YP_001001037.1), and SelD (YP_001001151.1) sequences from *C. jejuni*, and with *bona fide* MnmH (YP_003891307.1) and YedF (YP_002574971.1) sequences from *S. autotrophica* and *C. lari*, respectively. Identification of tRNA ^{Sec} (SelC) genes was based on tRNAScan-SE search. Genes are indicated by protein accession numbers or genomic coordinates. Blast e-values and tRNAscan-SE scores are reported. Unannotated or misannotated genes (see Fig. S7 and Table S2 for details) are shown in blue.

Table S2. Sec-encoding proteins identified in Epsilonproteobacteria genomes

Coding sequence	Protein[a]	blastx best hit	%id	Query Start	Query End	Sbj. Start	Sbj. End	Note	Selenoprotein identification
NC_002163.1:c1446341-1449145	FdhO	YP_002344890.1	100	1	2802	1	934		GenBank
NC_005090.1:694979-697810	FdhO	NP_906953.1	100	1	2829	1	943		GenBank
NC_007575.1:c850305-853118	FdhO	YP_393335.2	100	16	2811	1	932		GenBank
NC_009850.1:c1505948-1508749	FdhO	YP_001490422.1	100	1	2799	1	933		GenBank
NC_012039.1:330854-333676	FdhO	YP_002574968.1	100	1	2820	1	940		GenBank
NC_015674.1:1254832-1257606	FdhO	YP_004608051.1	100	1	2772	12	935		GenBank
NC_004917.1:c217811-220633	FdhO	NP_859759.1	100	577	2820	1	748	5' truncated	dbTEU[b] / bSECISearch[c]
	FdhO	NP_859760.1	100	1	552	1	184	3' truncated	
NC_008599.1:c1514186-1517143	FdhO	YP_892675.1	100	586	2955	1	790	5' truncated	dbTEU[b] / bSECISearch[c]
NC_009714.1:381285-384239	FdhO	YP_002574968.1	68	43	2952	1	940	missing	dbTEU[b] / bSECISearch[c]
NC_009715.1:c1695733-1698711	FdhO	YP_001408941.1	100	589	2976	1	796	5' truncated	dbTEU[b] / bSECISearch[c]
NC_009715.1:c745343-747622	FdhH	YP_001467138.1	83	520	2277	1	585	missing	dbTEU[b] / bSECISearch[c]
NC_009802.1:1290428-1292704	FdhH	YP_001467138.1	100	520	2274	1	585	5' truncated	dbTEU[b] / bSECISearch[c]
	FdhH	YP_001467137.1	100	1	420	1	140	3' truncated	
NC_009802.1:1622807-1625635	FdhO	YP_001467445.1	100	610	2826	1	739	5' truncated	dbTEU[b] / bSECISearch[c]
NC_009802.1:301772-304747	FdhO	YP_001466213.1	100	583	2973	1	797	5' truncated	dbTEU[b] / bSECISearch[c]
NC_009802.1:c1616627-1618870	FdhH	YP_001467440.1	100	1	420	1	140	3' truncated	dbTEU[b] / bSECISearch[c]
	FdhH	YP_001467439.1	100	472	2241	1	590	5' truncated	
NC_012115.1:1203781-1206606	FdhO	YP_002607700.1	100	601	2823	1	741	5' truncated	dbTEU[b] / bSECISearch[c]
	FdhO	YP_002607699.1	100	1	549	1	183	3' truncated	
NC_013512.1:806764-809622	FdhO	YP_003303872.1	100	610	2856	1	749	5' truncated	dbTEU[b] / bSECISearch[c]
	FdhO	YP_003303871.1	100	1	558	1	186	3' truncated	
NC_013512.1:c2082230-2085031	FdhO	YP_003305122.1	100	1	501	22	188	3' truncated	dbTEU[b] / bSECISearch[c]
	FdhO	YP_003305121.1	100	553	2799	1	749	5' truncated	
NC_014166.1:c957134-959935	FdhO	YP_003655137.1	100	1	543	1	181	5' truncated	dbTEU[b] / bSECISearch[c]
	FdhO	YP_003655136.1	100	595	2799	1	735	3' truncated	
NC_014166.1:c964208-967054	FdhO	YP_003655143.1	100	583	2844	1	754	5' truncated	dbTEU[b] / bSECISearch[c]
	FdhO	YP_003655144.1	100	1	558	1	186	3' truncated	
NC_014810.2:127304-130078	FdhO	YP_004072834.1	100	541	2772	1	744	5' truncated	dbTEU[b] / bSECISearch[c]
	FdhO	YP_004072833.1	100	1	489	12	174	3' truncated	
NC_017735.1:1130767-1133580	FdhO	YP_006221291.1	100	580	2811	1	744	5' truncated	dbTEU[b] / bSECISearch[c]
	FdhO	YP_006221290.1	100	1	528	1	176	3' truncated	
NC_017737.1:1409067-1411895	FdhO	YP_006223203.1	100	595	2826	1	744	5' truncated	dbTEU[b] / bSECISearch[c]
	FdhO	YP_006223202.1	100	16	543	1	176	3' truncated	
NC_017761.1:599104-601923	FdhO	YP_006234982.1	100	577	2817	1	747	5' truncated	dbTEU[b] / bSECISearch[c]
NC_018002.1:834797-837655	FdhO	YP_006403736.1	100	610	2856	1	749	5' truncated	dbTEU[b] / bSECISearch[c]
	FdhO	YP_006403735.1	100	1	558	1	186	3' truncated	
NC_018002.1:c2174715-2177576	FdhO	YP_006405067.1	100	1	558	1	186	3' truncated	dbTEU[b] / bSECISearch[c]
	FdhO	YP_006405066.1	100	610	2859	1	750	5' truncated	
NC_012115.1:c1187216-1188259	SelD	YP_002607680.1	100	1	1041	1	347		GenBank
NC_014935.1:299621-300664	SelD	YP_004167329.1	100	10	1041	1	344		GenBank
NC_017737.1:c1260015-1261040	SelD	YP_006223057.1	100	82	1023	1	314	5' truncated	dbTEU[b] / bSECISearch[c]
NC_017735.1:c1168443-1169468	SelD	YP_006221319.1	100	82	1023	1	314	5' truncated	dbTEU[b] / bSECISearch[c]
NC_014166.1:976595-977629	SelD	YP_003655157.1	100	58	1032	1	325	5' truncated	dbTEU[b] / bSECISearch[c]
NC_009802.1:1726587-1727609	SelD	YP_001467548.1	100	277	1020	1	248	5' truncated	dbTEU[b] / bSECISearch[c]
NC_007575.1:861177-862211	SelD	YP_393344.1	100	58	1032	1	325	5' truncated	dbTEU[b] / bSECISearch[c]
NC_004917.1:c1658852-1659898	SelD	NP_861265.1	100	154	1044	1	297	5' truncated	dbTEU[b] / bSECISearch[c]
NC_002163.1:c1439168-1440190	SelD	YP_002344883.1	100	97	1020	1	308	5' truncated	dbTEU[b] / bSECISearch[c]
NC_017761.1:c901529-902617	SelD	YP_006235254.1	100	112	1086	1	325	5' truncated	dbTEU[b] / bSECISearch[c]
NC_015674.1:c790126-791136	SelD	YP_004607535.1	100	52	1008	1	319	5' truncated	dbTEU[b] / bSECISearch[c]
NC_014810.2:108644-109654	SelD	YP_004072817.1	100	52	1008	1	319	5' truncated	dbTEU[b] / bSECISearch[c]
NC_012039.1:335663-336694	SelD	YP_002574972.1	100	70	1029	1	320	5' truncated	dbTEU[b] / bSECISearch[c]
NC_008599.1:308394-309425	SelD	YP_891542.1	100	79	1029	1	317	5' truncated	dbTEU[b] / bSECISearch[c]
NC_013949.1:c958499-959524	SelD	YP_003516809.1	100	118	1023	1	302	5' truncated	dbTEU[b] / bSECISearch[c]
NC_009715.1:c1656276-1657316	SelD	YP_891542.1	61	73	1005	2	309	missing	dbTEU[b] / bSECISearch[c]
NC_014166.1:c949137-949394	SelW-like	YP_003655126.1	100	1	255	1	85		GenBank
NC_002163.1:c671511-671753	SelW-like	YP_002575469.1	49	234	1	1	78	missing	dbTEU[b] / bSECISearch[c]
NC_008599.1:1234401-1234658	SelW-like	YP_003655126.1	32	16	237	1	75	missing	dbTEU[b]
NC_008599.1:1234830-1235069	SelW-like	YP_003655126.1	37	1	237	1	81	missing	dbTEU[b] / bSECISearch[c]
NC_009715.1:c589810-590067	SelW-like	YP_003655126.1	42	1	237	1	78	missing	dbTEU[b] / bSECISearch[c]
NC_009802.1:c1487351-1487608	SelW-like	YP_003655126.1	43	1	237	1	78	missing	dbTEU[b] / bSECISearch[c]
NC_012039.1:854195-854434	SelW-like	YP_002575469.1	97	234	1	1	78	wrong frame	dbTEU[b] / bSECISearch[c]
NC_014935.1:c600388-600780	Tr	YP_004167611.1	100	1	390	1	130		GenBank
NC_009715.1:1801466-1801996	Perox	YP_001409037.1	100	1	525	1	175	3' truncated	tblastn[d] / bSECISearch[c]
NC_012039.1:1300804-1300998	DUF466	YP_002575916.1	100	1	189	1	63	3' truncated	cmsearch / tblastn[e]
NC_004917.1:1706005-1706244	DUF466	NP_861314.1	100	1	234	1	78	3' truncated	cmsearch / tblastn[e]
NC_017761.1:c1129343-1129546	DUF466	YP_003304245.1	46	64	198	19	65	missing	cmsearch / tblastn[e]
NC_002163.1:c852178-852378	DUF466	YP_002344314.1	100	1	195	1	65	3' truncated	tfastx[f]
NC_008599.1:c58283-58486	DUF466	YP_891281.1	100	1	198	1	66	3' truncated	tfastx[f]

[a]Protein definition based on homology with dbTEU or GenBank. Distinction between formate dehydrogenase H (FdhH) and formate dehydrogenase O (FdhO) was based on the phylogenetic relation with known *E. coli* proteins.

[b]Identified by homology (tblastn) with U-containing proteins in dbTEU

[c]Optimal SECIS element identified by bSECISearch

[d]Identified by tblastn search with U-containig ORFs translated from complete genomes

[e]Identified by the SECIS covariance model followed by a tblastn search with full translations

[f]Identified by homology (tfastx36 -m BB) with U-containing ORFs of the same family

Table S3. Position of Sec-encoding codons and equivalent Cys-encoding codons in epsilonproteobacterial genomes[a]

Species	Accession	Fdh	SelD	SelW-like	Tr	Perox	DUF466
Arcobacter butzleri RM4018	NC_009850.1	-U1508206	+C1523183			+C561644	-C742321
Arcobacter nitrofigilis DSM 7299	NC_014166.1	-U959392<br>-U966496	+U976640	-U949364		+C781750	
Arcobacter sp. L	NC_017192.1	-C1428915				+C683355	
Campylobacter concisus 13826	NC_009802.1	+U302303<br>+U1290848<br>-U1618450<br>+U1623365	+U1726635	-U1487578		+C1921514	
Campylobacter curvus 525.92	NC_009715.1	-U747202<br>-U1698174	-U1657268	-U590037		-U1801471	
Campylobacter fetus subsp. fetus 82-40	NC_008599.1	-U1516609	+U308451	+U1234446<br>+U1234860		-C1737280	-U58288
Campylobacter hominis ATCC BAA-381	NC_009714.1	+U381861	-C1700243				
Campylobacter jejuni NCTC 11168	NC_002163.1	-U1448605	-U1440142	-U671723		+C731918	-U852183
Campylobacter lari RM2100	NC_012039.1	+U331379	+U335711	+U854225		+C745718	+U1300993
Helicobacter acinonychis str. Sheeba	NC_008229.1						
Helicobacter bizzozeronii CIII-1	NC_015674.1	+U1255321	-U791097				
Helicobacter cetorum MIT 00-7128	NC_017737.1	+U1409610	-U1260998				
Helicobacter cetorum MIT 99-5656	NC_017735.1	+U1131295	-U1169426				
Helicobacter cinaedi PAGU611	NC_017761.1	+U599656	-U902575				-U1129348
Helicobacter felis ATCC 49179	NC_014810.2	+U127793	+U108683				
Helicobacter hepaticus ATCC 51449	NC_004917.1	-U220081	-U1659856				+U1706239
Helicobacter mustelae 12198	NC_013949.1		-U959479				
Helicobacter pylori 26695	NC_000915.1						
Helicobacter pylori B8	NC_014256.1						
Helicobacter pylori ELS37	NC_017063.1						
Helicobacter pylori G27	NC_011333.1						
Helicobacter pylori Gambia94/24	NC_017371.1						
Helicobacter pylori India7	NC_017372.1						
Helicobacter pylori P12	NC_011498.1						
Helicobacter pylori PeCan4	NC_014555.1						
Helicobacter pylori Shi417	NC_017739.1						
Helicobacter pylori SouthAfrica7	NC_017361.1						
Nautilia profundicola AmH	NC_012115.1	+U1204330	-U1188205				
Nitratifractor salsuginis DSM 16511	NC_014935.1	+C668728	+U299675		-U600633		
Nitratiruptor sp. SB155-2	NC_009662.1	+C1457780			-C1134061		
Sulfuricurvum kujiense DSM 16994	NC_014762.1	+C206464			+C1029676		
Sulfurimonas autotrophica DSM 16294	NC_014506.1	+C718915	-C230256		+C2039345		
Sulfurimonas denitrificans DSM 1251	NC_007575.1	-U852563	+U861222				
Sulfurospirillum barnesii SES-3	NC_018002.1	+U835355<br>-U2177018	-C2156414			+C1892236	
Sulfurospirillum deleyianum DSM 6946	NC_013512.1	+U807322<br>-U2084530	-C2063754				+C1204375
Sulfurovum sp. NBC37-1	NC_009663.1	+C1555053	-C1881842		+C718488		
Wolinella succinogenes DSM 1740	NC_005090.1	+U695540	-C962320				

[a]Absolute position in the genomic sequence of U-encoding codons in the identified selenoproteins and of C-encoding codons in homologous sequences; the + and – symbols are used to indicate the direct and complementary strands, respectively.

Table S4. Conservation of SelA residues involved in hydrogen-bonded intra-dimer interactions

		Sec-decoding Helicobacteriaceae[b]			non-Sec-decoding Helicobacteriaceae[b]		
Position[a]	Residue	Consurf grade	dN/dS[c]		Consurf grade	dN/dS[c]	
68	N	6*	0.05 ±	0.022	4*	0.113 ±	0.24
70	K	2*	0.984 ±	0.293	6*	0.145 ±	0.298
72	V	6*	0.055 ±	0.068	7	0.094 ±	0.202
79	V	6	0.058 ±	0.091	9	0.077 ±	0.158
83	N	9	0.05 ±	0.002	1	2.929 ±	1.92
84	L	7	0.051 ±	0.03	5*	0.09 ±	0.191
85	G	9	0.05 ±	0.001	9	0.138 ±	0.285
87	A	8	0.05 ±	0.005	7	0.169 ±	0.345
89	L	4*	0.053 ±	0.052	6*	0.091 ±	0.194
101	A	6*	0.05 ±	0.016	4*	0.139 ±	0.289
104	Y	7	0.05 ±	0.005	3*	0.14 ±	0.29
105	S	3*	0.735 ±	0.477	5*	0.076 ±	0.155
106	N	7	0.059 ±	0.097	1	1.075 ±	0.489
108	E	9	0.05 ±	0.001	9	0.074 ±	0.151
111	L	9	0.05 ±	0.005	8	0.135 ±	0.281
119	R	9	0.05 ±	0.002	9	0.149 ±	0.306
122	H	7	0.05 ±	0.006	5*	0.125 ±	0.262
129	E	6	0.051 ±	0.035	3*	0.739 ±	0.608
140	N	9	0.05 ±	0.002	9	0.072 ±	0.143
141	N	9	0.05 ±	0.002	9	0.083 ±	0.175
151	N	7	0.089 ±	0.193	9	0.087 ±	0.184
155	E	1	1.128 ±	0.34	2*	0.874 ±	0.843
285	K	9	0.05 ±	0.001	9	0.077 ±	0.159
291	Q	9	0.05 ±	0.001	9	0.145 ±	0.298
312	R	9	0.05 ±	0.002	9	0.079 ±	0.164
315	R	9	0.05 ±	0.001	9	0.097 ±	0.207
318	K	9	0.05 ±	0.001	9	0.076 ±	0.156
325	E	1	1.042 ±	0.212	2*	0.092 ±	0.197
412	R	7	0.05 ±	0.012	4*	0.139 ±	0.289

[a]Residue position relative to the AaSelA sequence
[b]based on alignments of SelA coding sequences from Helicobacteriaceae with or without the Sec-decoding trait (see Fig. 3)
[c]dN/dS variations among sites calculated with PAML
*Below the confidence cut-off (less than 6 non-gaped homologue sequences)

Table S5. Conservation of SelA residues involved in hydrogen-bonded dimer-dimer interactions

		Sec-decoding Helicobacteriaceae[b]			non-Sec-decoding Helicobacteriaceae[b]		
Position[a]	Residue	Consurf grade	dN/dS[c]		Consurf grade	dN/dS[c]	
163	R	8	0.05 ±	0.008	4*	0.103 ±	0.22
166	L	9	0.05 ±	0.004	8	0.14 ±	0.291
168	E	9	0.05 ±	0.001	4*	-	
174	R	9	0.05 ±	0.001	3*	0.484 ±	0.491
188	E	7	0.05 ±	0.021	3*	0.142 ±	0.294
192	T	8	0.05 ±	0.01	1	3.112 ±	1.915
193	N	9	0.05 ±	0.002	2*	1.096 ±	0.544
196	K	7	0.05 ±	0.001	3*	0.103 ±	0.22
199	D	9	0.05 ±	0.001	9	0.07 ±	0.138
218	N	9	0.05 ±	0.002	6*	-	
220	Y	4*	0.075 ±	0.156	6*	-	
222	E	1	1.133 ±	0.397	6*	-	
224	F	9	0.05 ±	0	9	0.092 ±	0.197

[a]Residue position relative to the AaSelA sequence
[b]based on alignments of SelA coding sequences from Helicobacteriaceae with or without the Sec-decoding trait (see Fig. 3)
[c]dN/dS variations among sites calculated with PAML
*Below the confidence cut-off (less than 6 non-gaped homologue sequences)
